# FBXO22 promotes leukemogenesis by targeting BACH1 in MLL-rearranged acute myeloid leukemia

**DOI:** 10.1186/s13045-023-01400-0

**Published:** 2023-02-11

**Authors:** Xiao-Na Zhu, Yu-Sheng Wei, Qian Yang, Hao-Ran Liu, Zhe Zhi, Di Zhu, Li Xia, Deng-Li Hong, Yun Yu, Guo-Qiang Chen

**Affiliations:** 1 Institute of Aging & Tissue Regeneration, State Key Laboratory of Oncogenes and Related Genes and Chinese Academy of Medical Sciences Research Unit (No. 2019RU043), Ren-Ji Hospital, Shanghai Jiao Tong University School of Medicine (SJTU-SM), Shanghai, China; 2 Key Laboratory of Cell Differentiation and Apoptosis of Chinese Ministry of Education, Rui-Jin Hospital, SJTU-SM, Shanghai, China

**Keywords:** FBXO22, Leukemia stem cells (LSCs), Acute myeloid leukemia (AML), BTB and CNC homology 1 (BACH1)

## Abstract

**Background:**

Selectively targeting leukemia stem cells (LSCs) is a promising approach in treating acute myeloid leukemia (AML), for which identification of such therapeutic targets is critical. Increasing lines of evidence indicate that FBXO22 plays a critical role in solid tumor development and therapy response. However, its potential roles in leukemogenesis remain largely unknown.

**Methods:**

We established a mixed lineage leukemia (MLL)-AF9-induced AML model with hematopoietic cell-specific FBXO22 knockout mice to elucidate the role of FBXO22 in AML progression and LSCs regulation, including self-renewal, cell cycle, apoptosis and survival analysis. Immunoprecipitation combined with liquid chromatography-tandem mass spectrometry analysis, Western blotting and rescue experiments were performed to study the mechanisms underlying the oncogenic role of FBXO22.

**Results:**

FBXO22 was highly expressed in AML, especially in MLL-rearranged (MLLr) AML. Upon FBXO22 knockdown, human MLLr leukemia cells presented markedly increased apoptosis. Although conditional deletion of *Fbxo22* in hematopoietic cells did not significantly affect the function of hematopoietic stem cells, MLL-AF9-induced leukemogenesis was dramatically abrogated upon *Fbxo22* deletion, together with remarkably reduced LSCs after serial transplantations. Mechanistically, FBXO22 promoted degradation of BACH1 in MLLr AML cells, and overexpression of BACH1 suppressed MLLr AML progression. In line with this, heterozygous deletion of BACH1 significantly reversed delayed leukemogenesis in *Fbxo22*-deficient mice.

**Conclusions:**

FBXO22 promotes MLLr AML progression by targeting BACH1 and targeting FBXO22 might be an ideal strategy to eradicate LSCs without influencing normal hematopoiesis.

**Supplementary Information:**

The online version contains supplementary material available at 10.1186/s13045-023-01400-0.

## Introduction

Acute myeloid leukemia (AML), a class of highly prevalent and aggressive hematologic malignancies, is characterized by uncontrolled expansion of immature myeloid cells coupled with a differentiation blockage [1]. The development of AML is associated with accumulation of acquired genetic and epigenetic changes in hematopoietic stem/progenitor cells (HSPCs) [2]. Subsequent acquisitions of additional genetic and epigenetic aberrations in pre-leukemic HSPCs eventually give rise to leukemia stem cells (LSCs), which are responsible for leukemia initiation, progression, relapse and drug resistance [3, 4]. Chromosomal translocation of the mixed lineage leukemia (*MLL*) gene is recurrent alterations associated with aggressive and drug-resistant leukemia [5]. The t(9;11)(p22;q23) reciprocal translocation results in the expression of MLL-AF9 fusion gene and myelo-monoblastic AML associated with extramedullary tumor infiltration, frequent relapses and poor survival [6]. Despite advances in understanding the molecular basis of AML development, over 70% of AML patients cannot survive more than 5 years [7, 8]. Thus, identification of specific therapeutic targets for eliminating LSCs is an urgent clinical need for drug development.

The SCF (SKP1/CUL1/F-box) complex, the best-characterized cullin-RING ligases (CRLs) among ubiquitin ligases, consists of scaffold protein cullin 1 (CUL1), RBX1 (RING-box protein 1), adaptor protein SKP1 (S-phase kinase-associated protein 1) and F-box protein, the latter being a substrate recognition receptor to determine the substrate specificity of the SCF complex [9]. There are 69 putative F-box proteins in human, which are divided into three subclasses depending on the specific substrate recognition domains, FBXW (F-box and WD40 domain), FBXL (F-box and leucine-rich repeat) and FBXO (F-box only) proteins [10]. Accumulated lines of evidence showed that F-box proteins are promising targets for cancer therapy, including hematopoietic malignancies. For instances, FBXW7 deletion induced T-cell acute lymphoblastic leukemia (T-ALL) development without the requirement of other tumor-promoting factors [11], while it eliminated LSCs of chronic myeloid leukemia (CML) by preventing quiescence [12]. FBXL2, FBXL10 and FBXW11 had been linked to lymphocytic leukemia cell proliferation [13–15]. The overexpressed FBXO9 caused increased proliferation and survival in multiple myeloma (MM) [16], while loss of FBXO9 promoted AML development [17]. Also, FBXW4 was highly expressed and associated with poor survival in AML [18].

FBXO22 was believed to play a critical role in cancer development and therapy response [19]. The oncogenic role of FBXO22 was shown in colorectal, liver, lung and breast cancers [20–23], while the suppressive role of cancer metastasis was also been proposed in lung cancer and breast cancer [23, 24]. With these reports, several substrates of FBXO22 have been explored, such as lysine demethylase (KDM) 4A, KDM4B, BTB and CNC homology 1 (BACH1), phosphatase and tension homologue (PTEN), P53, liver kinase B (LKB1), which mediate the multiple functions in carcinogenesis [20–27]. However, the potential role of FBXO22 in leukemogenesis is not explored to date. Herein, we investigate the function of FBXO22 to MLL-AF9-induced AML development using two strains of hematopoietic-specific *Fbxo22* knockout mice and found that FBXO22 promotes leukemogenesis and contributes to LSC maintenance by targeting BACH1.

## Methods

### Mice

*Fbxo22*^*fl/fl*^ mice and C57BL/6 *Bach1* knockout mice were established at the Cyagen Biosciences Inc. *Fbxo22*^*fl/fl*^ mice were further crossed with Myxovirus resistance protein 1 *(Mx1)-Cre* or stem cell leukemia stem-cell enhancer *(Scl)-Cre* transgenic mice to achieve specifically inducible deletion of *Fbxo22* in hematopoietic cells. All these strains were maintained on a C57BL/6 background. To induce *Mx1-Cre* recombinase, polyinosinic–polycytidylic acid (pIpC, Sigma, 1 mg/ml in distilled water) was administered at 10 µg/g body weight every other day by intraperitoneal injection for 7 times; to induce *Scl-Cre* recombinase, tamoxifen (Sigma, 10 mg/ml in corn oil) was administered at 50 µg/g body weight daily by intraperitoneal injection for 21 days. All the animal experiments were approved by our institution and conducted according to the Guideline for Animal Care at Shanghai Jiao Tong University School of Medicine.

### MLL-AF9-induced murine AML model

A transplantable MLL-AF9-induced murine AML model was established as follows. Briefly, 10–12-week-old donor mice were injected with 5-Fluorouracil (5-FU) at 120 mg/kg body weight by intraperitoneal injection. Lin^−^ bone marrow (BM) cells were isolated at day 6 after 5-FU treatment and infected twice with retroviral pMIG-MLL-AF9-IRES-GFP supernatant in the presence of 4 µg/ml polybrene. Infected cells were cultured in StemSpan™ SFEM medium supplemented with 20 ng/ml mouse stem cell factor, 10 ng/ml mouse interleukin-3 (IL-3) and 10 ng/ml mouse interleukin-6 (IL-6). After the second round of infection, cells were collected and intravenously injected into lethally irradiated C57BL/6 mice at 2 × 10^5^ per recipient with 2 × 10^5^ competitor BM cells. Serial transplantations were performed with 1 × 10^4^ purified GFP^+^ BM leukemia cells or 1000 purified leukemic-granulocyte monocyte progenitor (LGMP) cells of either primary or secondary recipient mice mixed with 2 × 10^5^ competitor BM cells. For the limiting dilution analysis, the indicated *Fbxo22*^+/+^ and *Fbxo22*^−/−^ GFP^+^ BM cells collected from primary recipients were co-transplanted with 2 × 10^5^ competitor cells into lethally irradiated recipient mice. The survival times were recorded to calculate LSCs frequencies using L-Calc software from StemCell Technologies.

### Flow cytometry analysis and sorting

BM single cells suspension was freshly isolated and filtered through a 40 μm strainer. Peripheral blood (PB) cells were treated with ammonium chloride potassium (ACK) lysis buffer to remove red blood cells and resuspended with phosphate buffer saline (PBS). The cells were surface-stained using indicated fluorochrome-conjugated antibodies following the manufacturer’s instructions. Cell cycle distribution was examined by Hoechst 33342 and Ki-67-BV786 staining after surface-stained. For intracellular staining, cells were fixed and permeabilized by using BD Cytofix/Cytoperm Fixation/Permeabilization Kit following the manufacturer’s protocol. Apoptosis was analyzed with annexin-V-Allophycocyanin (APC) and 4',6-diamidino-2-phenylindole (DAPI) staining according to the manufacturer’s instructions. Detailed information of antibodies and dyes used in these experiments is listed in Additional file 1: Table S1. Cell sorting was performed using a BD Fluorescence Activating Cell Sorter (FACS) Aria II or Aria III after staining.

### Immunoprecipitation and mass spectrometry analysis

Immunoprecipitation of Flag-tagged proteins or endogenous proteins and Nano-LC-MS/MS with electrospray ionization used to identify interacting proteins were performed as previously described [28, 29]. The antibodies used in immunoprecipitation are listed in Additional file 1: Table S1.

### Denaturing ubiquitination assay

THP-1 cells were treated with 10 µM of the proteasome inhibitor MG132 for 6 h, harvested and lysed with denature lysis buffer (denatured IP buffer 50 mM Tris-HCl, pH 6.8, 2% SDS) by boiling at 100 °C for 20 min to dissociate protein–protein interactions. After centrifugation at 17,000 g for 10 min at 4 °C, 70 µl of supernatants was diluted by 1.2 ml 1 × RIPA buffer (50 mM Tris-HCl, PH 7.6, 150 mM NaCl, 1 mM EDTA, 1% NP-40, 1 mM PMSF and 1 × protease inhibitor cocktail) and subjected to immunoprecipitation with BACH1 antibody, followed by Western blot analysis to visualize polyubiquitylated protein bands.

### Colony-forming unit assays

BM cells isolated from *Fbxo22*^+/+^ and *Fbxo22*^−/−^ or *Scl-Cre*^−^;*Fbxo22*^*fl/fl*^ and *Scl-Cre*^+^;*Fbxo22*^*fl/fl*^ mice were plated into 12-well plates containing MethoCult M3434 (StemCell Technologies) for colony-forming unit-granulocyte, erythrocyte, monocyte and megakaryocyte (CFU-GEMM) or MethoCult M3534 (StemCell Technologies) for colony-forming unit-granulocyte and macrophage (CFU-GM) colony formation analysis at 2.5 × 10^4^ cells/well, MethoCult M3334 (StemCell Technologies) for colony-forming unit-erythrocyte (CFU-E) or MethoCult M3630 (StemCell Technologies) for colony-forming unit-pre-B lymphocyte (CFU-Pre-B) colony formation analysis at 2.5 × 10^5^ cells/well according to the manufacturer’s protocols. GFP^+^ or LGMP cells sorted from primary AML recipients were plated into 12-well plates containing complete MethoCult M3534 medium at indicated numbers according to the manufacturer’s instructions. For the colony-forming assay of THP-1, FBXO22-knockdown THP-1 cells or control cells were plated into 12-well plates containing complete MethoCult H4436 (StemCell Technologies) at 500 cells/well. For the colony-forming assay of AML patients’ mononuclear cells (MNCs), FBXO22-knockdown or overexpressed cells with control cells were plated into 24-well plates containing MethoCult H4436 at 40,000 cells/well. Colonies were imaged and counted 7–10 days after plating.

### Cell lines and cell culture

THP-1, MV4-11, Molm13, Kasumi-1, HL-60 and U937 cells were maintained in RPMI-1640 supplemented with 10% fetal bovine serum (FBS). 32D cells were maintained in RPMI-1640 with 10% FBS and 10 ng/mL mouse IL-3. 293 T cells were maintained in DMEM supplemented with 10% FBS. Cells were cultured in a humidified incubator at 37 °C with 5% CO_2_. All the cell lines were purchased from the American Type Culture Collection (ATCC).

### AML patients

Primary human AML MNCs were separated by density gradient centrifugation from BM samples of patients with AML from Ren-ji Hospital, Shanghai Jiao Tong University School of Medicine. Written informed consent was obtained from all the patients, and all the procedures were approved by the Ethics Committee for Medical Research (IRB) at Ren-ji Hospital. Samples were frozen in FBS with 10% dimethyl sulfoxide (DMSO) and stored in liquid nitrogen.

### Public databases analysis

Publicly annotated datasets from Gene Expression Omnibus (GEO), including GSE1159, GSE13159, GSE7186, GSE48173, GSE76008, GSE63720, GSE68172 and GSE138883, were downloaded and analyzed using RMA method by R package affyPLM (1.30.18) or using GEO2R independently. Gene expression data of AML samples from The Cancer Genome Atlas (TCGA) database were analyzed by R package TCGAbiolinks (2.25.3) followed with processed by log_10_, and public clinical annotations were downloaded from cBioportal (http://www.cbioportal.org/). Gene expression counts and clinical cytogenetic results of Beat-AML were explored through the interactive browsers, including Vizome (http://vizome.org/aml2/) and BeatAML2 (https://biodev.github.io/BeatAML2/). Proteomic database of AML (PXD032110) was accessed and queried through Proteomic and Phosphoproteomic Landscapes of AML (http://www.leylab.org/amlproteome).

### Plasmids, virus construction and infection

shRNAs targeting human FBXO22 or a normal control (NC) shNC were constructed using a lentiviral vector, pLKO.1-GFP (target sequences listed in Additional file 1: Table S1). FBXO22 coding sequence (CDS) was inserted into a doxycycline (Dox)-inducible lentiviral vector pINDUCER21 and a retroviral plasmid pQCXIN, respectively. Lentiviruses were produced using polyethylenimine (PEI) transfection method with the packaging plasmids pCMV-dR8.91 (Δ8.9) and pMDG. For rescue experiments, Flag-tagged FBXO22 CDS with multiple point mutations in shFBXO22#1 target sequence was inserted into the retroviral plasmid pQCXIN. HA-tagged Fbxo22 CDS was inserted into the retroviral plasmid pMIGR1-RFP. In order to study the effect of BACH1 on the progression of AML in human AML cell lines and animal models, Flag-tagged BACH1 CDS and HA-tagged Bach1 CDS were inserted into pQCXIN and pMIGR1-RFP, respectively. Retrovirus was produced using PEI transfection method with the packaging plasmids gag-pol and VSV-G in human cells or pCL-ECO in mouse cells. The lentiviral or retroviral supernatant was collected to infect cells by twice centrifugation at 1800 rpm for 90 min.

### Proliferation analysis in vitro

shRNAs targeting human FBXO22 or shNC lentiviral supernatant was used to infect several human leukemia cell lines THP-1, MV4-11 and human AML patients’ MNCs, followed by analysis for the proliferation ability in vitro at indicated time points. The cell counting kit 8 (CCK8) experiment for in vitro proliferation assay was performed using the CCK8 Kit (Dojindo) according to the manufacturer’s instructions. Human AML patients’ cells were cultured in Stemspan basic medium (StemCell Technologies) supplemented with 20 ng/ml human stem cell factor, 10 ng/ml human IL-3, and 10 ng/ml human IL-6. (All the growth factors were from PeproTech.)

### Homing

6 × 10^6^ *Fbxo22*^+/+^ and *Fbxo22*^−/−^ GFP^+^ BM cells collected from primary recipients were transplanted into lethally irradiated mice. The frequencies of GFP^+^ cells in the PB, spleen and BM cells were measured at 16 h after transplantation by flow cytometry.

### CRISPR-CAS9

gFBXO22 in the lentiviral vector pLenti-U6-gRNA-mCMV-SaCas9-P2A-sfGFP was purchased from Obio Technology (Shanghai, China). The target sequence is listed in Additional file 1: Table S1. THP-1 cells were infected with virus carrying CAS9 and gRNA for 48 h and planted into 96-well plates to obtain FBXO22 knockout clones.

### Western blot

Protein extracts were separated by sodium dodecyl sulfate–polyacrylamide gel electrophoresis (SDS-PAGE), transferred onto 0.45 μm nitrocellulose membrane (Millipore), blocked with 5% nonfat milk in PBS and sequentially incubated with the indicated primary antibodies followed by reacted with horseradish peroxidase (HRP)-linked secondary antibody (Cell Signaling, Beverly, MA); Immobilon Western Chemiluminescent HRP substrate kit (Merck Millipore) was used for detection.

### qRT-PCR

Total RNA was prepared using TRIzol (Invitrogen) and subjected to reverse transcription to synthesize cDNA using random primers (Takara) and M-MLV Reverse Transcriptase (Promega) following the manufacturer’s instructions. qRT-PCR was performed using the SYBR Green PCR Master Mix (Applied Biosystems). The experiments were conducted in triplicate with an Applied Biosystems QuantStudio5 PCR system. The mRNA level was normalized to the level of reference genes RNA transcripts. The primer sequences used are shown in Additional file 1: Table S1.

### Quantification and statistical analysis

For comparison between two experimental groups or a specific pair in multi-group, two-tailed unpaired Student’s *t* test was used, error bars denote mean ± SD. One-way ANOVA plus Fisher’s LSD test was using for comparisons involving more than two groups in datasets. For comparison of proliferation curves, two-way ANOVA was used. Pearson correlation coefficient was used to analyze the correlation between two continuous variables. All statistical analyses were conducted using Prism 8 and SPSS 20.0. Differences were considered significant at *P* < 0.05.

## Results

### FBXO22 is highly expressed in human AML and required for cell growth of AML especially MLLr AML

**Fig. 1:**
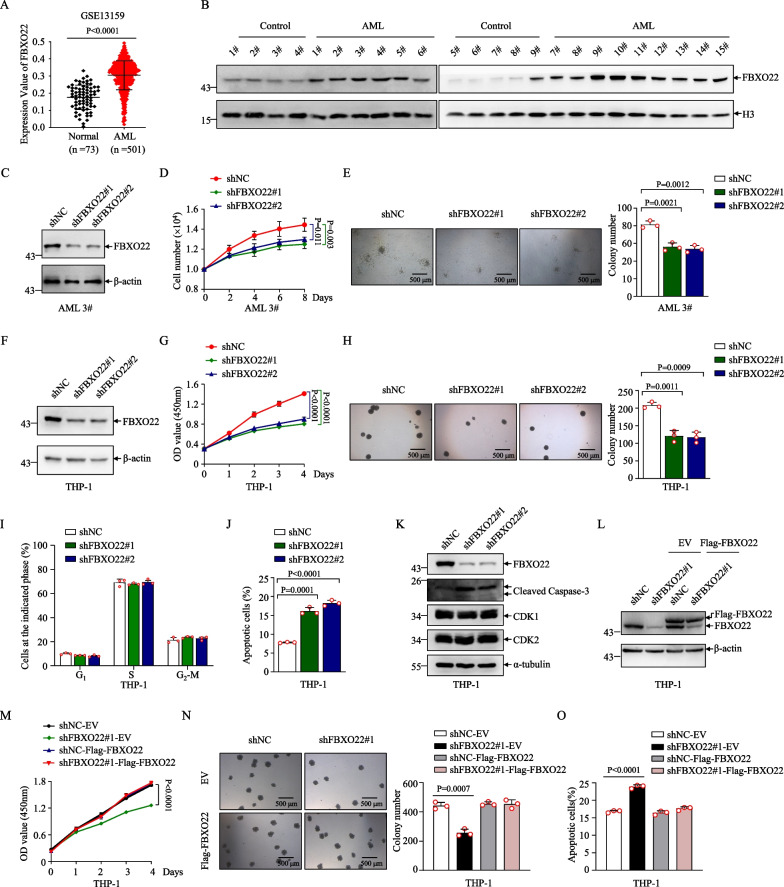
Highly expressed FBXO22 is required for the growth of AML especially MLLr AML cells. **A** FBXO22 mRNA expression level was analyzed in GSE13159 database. **B** FBXO22 protein in BM mononuclear cells (BMMC) of AML (including 1 case of M1 or M2, 9 cases of M4 and 4 cases of M5 AML samples) and non-leukemic individuals was analyzed by Western blot with Histone 3 (H3) as loading control. **C,** **D** AML 3# MNCs were infected with shFBXO22#1, shFBXO22#2 and shNC, and FBXO22 protein was immunoblotted with β-actin as loading control (**C**). Cell numbers were counted at the indicated days (**D**). **E** Colony-forming assay (left) for AML 3# MNCs infected with shNC, shFBXO22#1 and shFBXO22#2, and colony numbers at day 10 were counted (right, *n* = 3). **F**, **G** THP-1 cells were infected with shFBXO22#1, shFBXO22#2 and shNC, and FBXO22 protein was immunoblotted with β-actin as loading control (**F**), and cell proliferation was examined by CCK-8 assay at the indicated days (**G**). **H** Colony-forming assay (left) for THP-1 cells infected with shNC, shFBXO22#1 and shFBXO22#2, and colony numbers at day 7 were counted (right, *n* = 3). **I**–**K** Cell-cycle (**I**) or apoptosis (**J**) analysis for THP-1 cells infected with shNC, shFBXO22#1 and shFBXO22#2 (*n* = 3). Indicated proteins were immunoblotted with α-tubulin as loading control (**K**). **L**, **M** THP-1 cells infected with shNC or shFBXO22#1 were stably transfected with EV or Flag-tagged shRNA-resistant FBXO22, and indicated proteins were immunoblotted with β-actin as loading control (**L**), and cell proliferation was examined by CCK-8 assay at the indicated days (**M**). **N** Colony-forming assay (left) for the indicated THP-1 cells, and colony numbers at day 7 after plating were counted (right, *n* = 3). **O** Apoptosis analysis for indicated THP-1 cells (*n* = 3). Error bars denote mean ± SD. Statistical significance was determined by two-tailed unpaired *t* test (**A**, **E**, **H**–**J** and **N**–**O**) or two-way ANOVA (**D**, **G** and **M**), and the *P* values were shown. All experiments were repeated three times with similar results

By analyzing the mRNA expression levels of F-box proteins in a larger AML patient cohort (GSE1159), we found that FBXO22 had higher expression in human AML than that in healthy samples, and ranked among the top candidates in all F-box proteins available in this cohort (Additional file 2: Fig. S1A), which was also supported by three other cohorts from AML patients (GSE13159, GSE7186 and GSE48173) (Fig. 1A and Additional file 2: Fig. S1B). We also analyzed FBXO22 protein levels in mononuclear cells from BM of 15 cases of various morphologic subtypes of primary human AML together with samples from 9 cases of non-leukemic individuals as control (Additional file 1: Table S1), and results revealed that FBXO22 proteins were also aberrantly elevated to various degrees in AML patients compared to control individuals (Fig. 1B). Of note, the control group 9#, who had medication history of platelet lifting capsule (Additional file 1: Table S1), also presented highly expressed FBXO22. Together, these data suggest that FBXO22 might play a role in AML.

To determine the potential role in primary AML, we knocked down FBXO22 in BM mononuclear cells from 3# patient, who had higher FBXO22 protein (Fig. 1B), by two pairs of short hairpin RNAs (shRNAs) specifically against FBXO22 with the scramble shRNA (shNC) as a control. The results revealed that both shRNAs markedly deleted FBXO22 expression (Fig. 1C), and FBXO22 knockdown inhibited cell growth and clonogenicity in the primary AML cells (Fig. 1D, E). Reciprocally, transfected overexpression of Flag-FBXO22 in BM mononuclear cells from 6# patient, who had relatively lower FBXO22 protein (Fig. 1B), promoted the primary AML cell growth and colony formation (Additional file 2: Fig. S1C–E).

To explore whether FBXO22 expression is associated with AML subtypes, we analyzed FBXO22 expression by one-way ANOVA followed by Fisher’s LSD test in TCGA-LAML and Beat-AML cohort [30]. The results showed that FBXO22 was expressed at a higher level in AML patients harboring t(11q23)/MLL in comparison with AML subtypes with t(8;21), t(15;17), inv(16)/t(16;16), inv(3)/t(3;3), complex cytogenetics and other cytogenetics except for AML with normal karyotype in TCGA-LAML cohort (Additional file 2: Fig. S1F) and Beat-AML cohort (Additional file 2: Fig. S1G). These data suggested that FBXO22 was highly expressed in t(11q23)/MLL AML patients compared with most AML subtypes with other abnormal cytogenetic alteration. In line, higher FBXO22 mRNA and protein levels were detected in MLLr AML cell lines (Molm13, THP-1 and MV4-11) than non-MLLr cell lines (Kasumi-1, HL-60 and U937) (Additional file 2: Fig. S1H, I). To further examine the potential role of FBXO22 in MLLr AML, we knocked down FBXO22 expression in MLLr AML cell line THP-1 (Fig. 1F) and found that FBXO22 knockdown resulted in a markedly inhibition of cell growth and clonogenicity in THP-1 cells (Fig. 1G, H). In addition, FBXO22 knockdown significantly promoted apoptosis with caspase-3 activation of THP-1 cells, while had no effects on cell cycle (Fig. 1I–K). Furthermore, the re-expression of shRNA-resistant Flag-tagged FBXO22 in FBXO22-knockdown THP-1 cells completely rescued the defects in cellular growth, clonogenic ability and apoptosis caused by FBXO22 knockdown in THP-1 cells (Fig. 1L–O), supporting the specificity of the shRNAs against FBXO22. Of note, overexpression of FBXO22 in THP-1 cells did not affect proliferation/colony forming (Fig. 1M–N), which might be due to higher basal expression of FBXO22 in THP-1 cells. Collectively, these data suggest that FBXO22 is required for maintaining survival of human MLLr AML cells.

### Deletion of *Fbxo22* fails to affect normal hematopoiesis

**Fig. 2:**
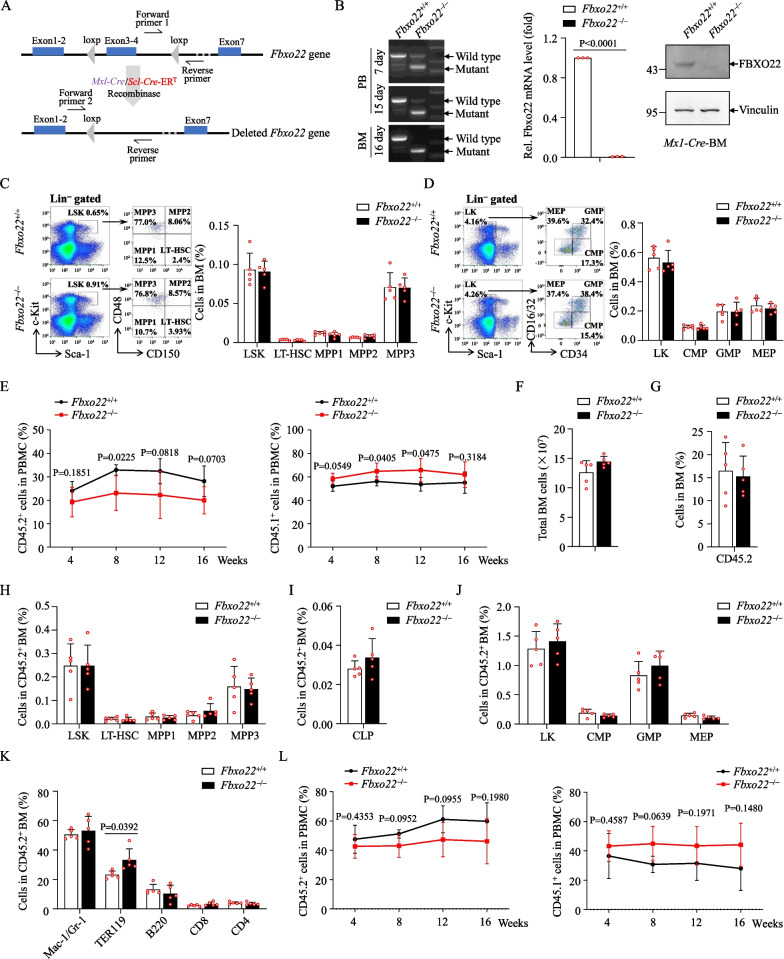
Deletion of *Fbxo22* fails to affect normal hematopoiesis. **A** Schematic of the *Fbxo22* floxed allele showing the deletion of floxed exons 3–4 following Cre recombinase activity. Use of *Mx1-Cre* or *Scl-Cre*-ER^T^ results in specific deletion in HSCs following pIpC or tamoxifen treatment. **B** *Fbxo22* deletion was evaluated by genotyping in PBMC and BM at indicated times after pIpC treatment (left), qRT-PCR (middle) and Western blot (right) in total BM cells at day 21 after pIpC treatment in mice. **C**, **D** Representative flow cytometry plots (left) and the percentages of LSK cells (Lin^−^Sca-1^+^c-Kit^+^), LT-HSCs (Lin^−^Sca-1^+^c-Kit^+^CD150^+^CD48^−^), MPP1 (Lin^−^Sca-1^+^c-Kit^+^CD150^−^CD48^−^), MPP2 (Lin^−^Sca-1^+^c-Kit^+^CD150^+^CD48^+^), and MPP3 (Lin^−^Sca-1^+^c-Kit^+^CD150^−^CD48^+^) (**C**, right, *n* = 5) and LK cells (Lin^−^Sca-1^−^c-Kit^+^), CMPs (Lin^−^Sca-1^−^c-Kit^+^CD16/32^−^ CD34^+^), GMPs (Lin^−^Sca-1^−^c-Kit^+^CD16/32^+^CD34^+^) and MEPs (Lin^−^Sca-1^−^c-Kit^+^ CD16/32^−^CD34^−^) in BM from *Fbxo22*^+/+^ and *Fbxo22*^−/−^ mice (**D**, right, *n* = 5). (**E–K**) Primary competitive transplantation assay was conducted with *Fbxo22*^+/+^ and *Fbxo22*^−/−^ BM CD45.2 cells (1 × 10^6^) along with CD45.1 competitor cells (1 × 10^6^). Percentages of donor or recipient-derived PB cells at the indicated times (**E**), and the indicated cells were analyzed at 16 weeks (**F**–**K**, *n* = 5). **L** Primary transplantation was conducted with sorted LT-HSCs (1 × 10^3^) from *Fbxo22*^+/+^ and *Fbxo22*^−/−^ BM cells along with 1 × 10^5^ BM cells from CD45.1 competitor. Percentages of donor or recipient-derived cells in PB were analyzed at the indicated time points. Error bars denote mean ± SD. Statistical significance was determined by two-tailed unpaired *t* test. All animal experiments were repeated at least twice with similar results

To address whether FBXO22 is required for leukemogenesis, we crossed *Fbxo22*^*fl/fl*^ mice with an *Mx1-Cre* strain to produce *Mx1-Cre*;*Fbxo22*^*fl/fl*^ mice (Fig. 2A). Two weeks post-pIpC injection, efficient deletion of *Fbxo22* was confirmed by genotyping, qRT-PCR and Western blot (Fig. 2B). Hereafter, pIpC-treated *Mx1-Cre*^–^;*Fbxo22*^*fl/fl*^ and *Mx1-Cre*^+^;*Fbxo22*^*fl/fl*^ were referred as *Fbxo22*^+/+^ and *Fbxo22*^–/–^ mice, respectively. Considering that the *Mx1* promoter-driving Cre is expressed in both hematopoietic cells and stromal cells [31], we also crossed *Fbxo22*^*fl/fl*^ mice with a tamoxifen-induced HSPC specific *Scl-Cre* strain [32] to get *Scl-Cre*;*Fbxo22*^*fl/fl*^ mice (Fig. 2A), and the *Fbxo22* deficiency in *Scl-Cre*^+^;*Fbxo22*^*fl/fl*^ mice was also confirmed at 3 weeks post-tamoxifen treatment (Additional file 3: Fig. S2A).

Although FBXO22 overexpression had no effects on cell growth and colony formation ability in mouse normal progenitor 32D cells (Additional file 3: Fig. S2B–D), and FBXO22 presented the lower expression in healthy individuals compared with AML (Fig. 1B), we still investigated the possible influences of *Fbxo22* deletion on normal hematopoiesis and showed that *Fbxo22* depletion did not trigger significant changes in total BM cell number or frequencies of different subpopulations of HSCs, HSPCs and differentiated lineages in BM at 8 weeks after pIpC induction (Fig. 2C, D and Additional file 3: Fig. S2E–G). There was comparable proportion of apoptotic cells in *Fbxo22*^+/+^ and *Fbxo22*^–/–^ HSCs (Additional file 3: Fig. S2H). Meanwhile, the colony-forming unit (CFU) assay showed comparable proportion of CFU-GEMM, CFU-GM and CFU-pre-B, and a slightly higher proportion of CFU-E in *Fbxo22*^–/–^ mice (Additional file 3: Fig. S2I). The similar results could also be seen in *Scl-Cre*;*Fbxo22*^*fl/fl*^ mouse model (Additional file 3: Fig. S2J–O). These data indicate that *Fbxo22* deletion has no obvious impact on normal hematopoiesis at least on young mice. To further investigate the effect of *Fbxo22* deletion on the repopulation capacity of HSCs, we competitively transplanted BM cells from *Fbxo22*^+/+^ or *Fbxo22*^–/–^ mice (CD45.2^+^) mixed with competitor cells into lethally irradiated recipients (CD45.1^+^) and monitored the repopulation of donor-derived cells in PB at indicated times. As shown in Fig. 2E–K, no obvious difference was seen in total BM cell numbers, frequencies of donor-derived HSCs, HSPCs and differentiated lineages in BM cells from *Fbxo22*^+/+^ and *Fbxo22*^–/–^ recipients at 16 weeks post-transplantation. We also performed another competitive assay with the same numbers of long-term (LT)-HSCs from *Fbxo22*^+/+^ and *Fbxo22*^–/–^ mice, and similar results were observed in PB analysis at indicated times after transplantation (Fig. 2L). Together, FBXO22 does not significantly affect the function of normal HSCs.

### Loss of *Fbxo22* impairs MLL-AF9-induced mouse AML development during serial transplantation

**Fig. 3:**
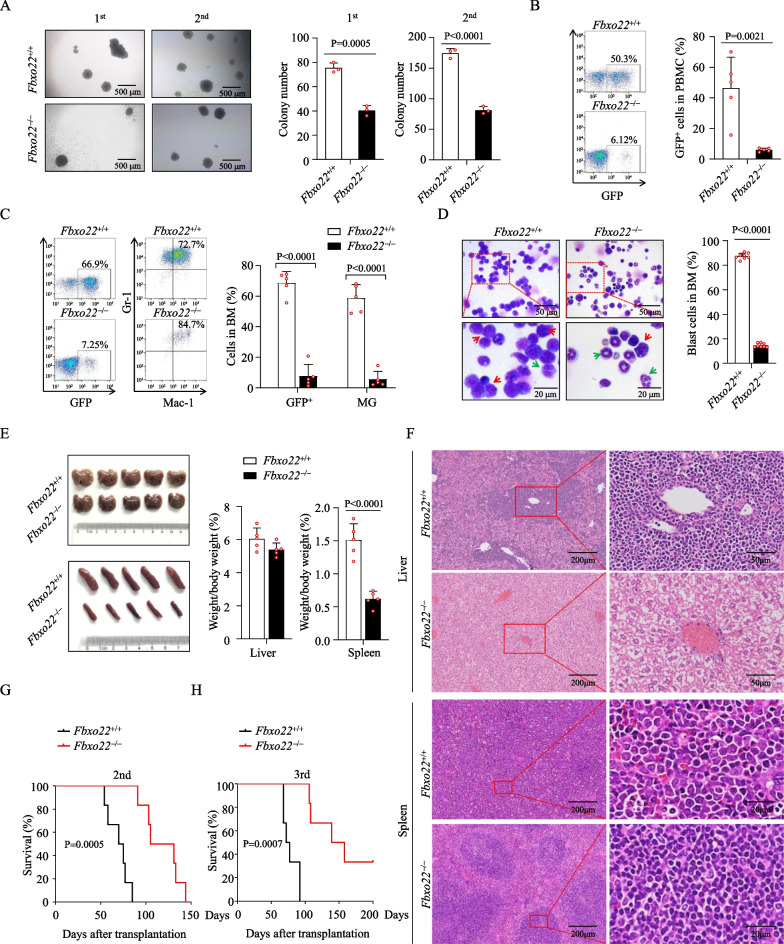
Loss of *Fbxo22* impairs MLL-AF9-induced mouse AML development during serial transplantation. **A** Representative images (left) of serial colonies and colony numbers (middle and right, *n* = 3) formed by GFP^+^ cells collected from the primary recipients. **B**, **C** Flow cytometry plots (left) and the percentage of GFP^+^ cells in PBMC (right, B, *n* = 5) and the percentages of GFP^+^ cells and MGs (GFP^+^Mac-1^+^Gr1^+^) in BM (right, **C**, *n* = 5) from recipients with secondary transplantation of AML cells. **D** Representative images of Giemsa-Wright staining (left) and frequencies of blast cells (right) for *Fbxo22*^+/+^ and *Fbxo22*^−/−^ BM cells upon the second transplantation. Red and green arrows point to representative blast cells and differentiated cells, respectively. **E**, **F** Gross pathology (left, **E**) and relative weights (right, **E**) and hematoxylin–eosin staining (**F**) of the livers and spleens from the secondary recipients (*n* = 5). **G**, **H** Survival curves for recipient mice receiving *Fbxo22*^+/+^ or *Fbxo22*^−/−^ MLL-AF9^+^ BM cells upon the second (**G**) and third transplantation (**H**) (*n* = 6). Error bars denote mean ± SD. Statistical significance was determined by two-tailed unpaired *t* test (**A**–**E**) or log-rank test (**G**, **H**), and the *P* values were shown. All animal experiments were repeated at least twice with similar results

Consistent with the high expression of FBXO22 in human MLLr AML cells, we demonstrated that FBXO22 was also highly expressed in mouse GFP^+^ leukemia cells from a MLL-AF9-induced AML model [33], as determined by Western blot (Additional file 4: Fig. S3A). Then, we used this transplantable murine AML model to explore the potential function of FBXO22 in AML development. Briefly, Lin^–^ HSPCs from BM of *Fbxo22*^+/+^ and *Fbxo22*^–/–^ mice were infected with MLL-AF9-GFP retrovirus and then transplanted into lethally irradiated recipients (Additional file 4: Fig. S3B). The results demonstrated that *Fbxo22* deficiency had no effect on GFP^+^ leukemia cells in PB of primary recipient mice at 5 weeks after transplantation, but slightly inhibited GFP^+^ myeloid leukemia cells in BM of these recipient mice (Additional file 4: Fig. S3C, D). The recipients of MLL-AF9-GFP-infected *Fbxo22*^–/–^ cells had also comparable survival time with recipients infected *Fbxo22*^+/+^ cells (Additional file 4: Fig. S3E). Consistently, we observed similar results in primary transplanted mice by MLL-AF9-GFP-infected Lin^–^ cells from *Scl-Cre*;*Fbxo22*^*fl/fl*^ mice (Additional file 4: Fig. S3F–H). Afterward, we performed serial CFU replating and serial transplantation assays with the same number of GFP^+^ AML cells. As shown in Fig. 3A, the *Fbxo22*^–/–^ AML cells had a remarkable reduction in CFUs on first and secondary plating. In line, *Fbxo22* deficiency significantly inhibited the engraftment of GFP^+^ myeloid leukemia cells in PB and BM of secondary recipient mice (Fig. 3B, C). Giemsa-Wright staining displayed a lower frequency of immature blast cells in BM, together with less massive splenomegaly and significantly decreased leukemia infiltration in spleen and liver in secondary recipients of *Fbxo22*^–/–^ AML cells (Fig. 3D–F). In line, recipient mice receiving *Fbxo22*^–/–^ leukemia cells showed remarkably prolonged survival times during secondary and tertiary transplantation (Fig. 3G, H). Similarly, the deficiency of *Fbxo22* in *Scl-Cre*;*Fbxo22*^*fl/fl*^ mice also significantly inhibited AML development during secondary transplantation (Additional file 5: Fig. S4A–D), and had markedly increased survival time in both secondary and tertiary transplantation (Additional file 5: Fig. S4E, F).

To exclude the possibility that the defective homing ability contributes to the effects of *Fbxo22* loss, GFP^+^ AML cells sorted from *Fbxo22*^+/+^ and *Fbxo22*^–/–^ recipients were injected into lethally irradiated mice, followed by detection of homed GFP^+^ AML cells in PB, spleen and BM. No significant difference was detected between two groups at 16 h after injection (Additional file 5: Fig. S4G). In addition, GFP^+^ AML cells from *Mx1-Cre*^–^;*Fbxo22*^*fl/fl*^ and *Mx1-Cre*^+^;*Fbxo22*^*fl/fl*^ mice were transplanted into lethally irradiated recipients, followed treatment with pIpC from day 7 to induce *Fbxo22* deletion (Additional file 5: Fig. S4H). Survival data indicated that acquired *Fbxo22* deletion still delayed the progression of AML and prolonged the survival of recipient mice (Additional file 5: Fig. S4I). Taken together, our data indicate that *Fbxo22* loss significantly represses development of AML.

### Loss of *Fbxo22* impairs the function of LSCs

**Fig. 4:**
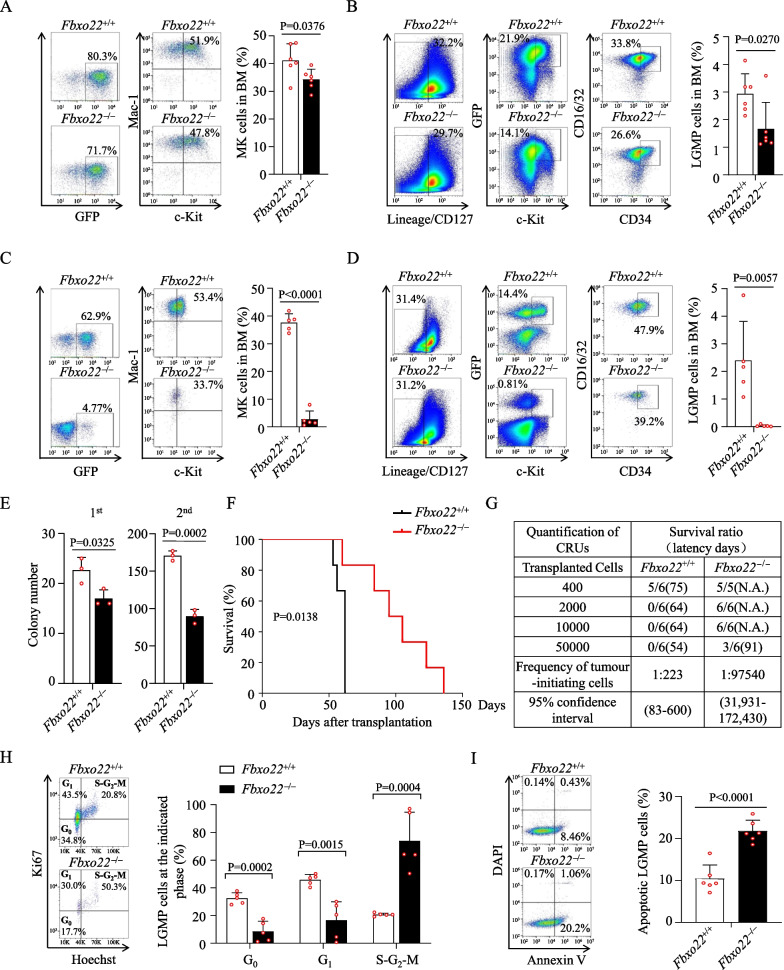
Loss of *Fbxo22* impairs the function of LSCs. **A**, **B** Flow cytometry plots (left) and the percentages of MKs (**A**) and LGMP (**B**) in BM from the primary transplanted recipients (right, *n* = 6). **C**, **D** Flow cytometry plots (left) and the percentages of MKs (**C**) and LGMP (**D**) in BM from the secondary transplanted recipients (right, *n* = 5). **E** Serial colonies and colony numbers (*n* = 3) formed by *Fbxo22*^+/+^ and *Fbxo22*^−/−^ LGMP cells collected from the primary recipients. **F** Survival curves for recipients receiving *Fbxo22*^+/+^ and *Fbxo22*^−/−^ LGMP cells upon secondary transplantation (*n* = 6). **G** Limiting dilution assays comparing the frequencies of LSCs in *Fbxo22*^+/+^ and *Fbxo22*^−/−^ MLL-AF9^+^ BM cells. The indicated GFP^+^ *Fbxo22*^+/+^ and *Fbxo22*^−/−^ MLL-AF9^+^ BM cells collected from primary recipients were co-transplanted with 2 × 10^5^ BM competitor cells into lethally irradiated recipients. The competitive repopulating units (CRUs) were calculated by L-Calc software. **H**, **I** Cell-cycle (**H**) or apoptosis (**I**) analysis of LGMP cells in BM from the secondary recipients transplanted with *Fbxo22*^+/+^ and *Fbxo22*^−/−^ AML cells (*n* = 5–6). Error bars denote mean ± SD. Statistical significance was determined by two-tailed unpaired *t* test (**A**–**E** and **H**, **I**) or log-rank test (**F**), and the *P* values were shown. All animal experiments were repeated at least twice with similar results

LSCs are believed to be responsible for drug resistance and relapse of AML patients [34]. Analysis on three datasets (GSE63270, GSE68172 and GSE138883) showed that the FBXO22 transcript level was higher in AML LSCs than that in HSCs (Additional file 6: Fig. S5A). A 17-gene LSC score (LSC17) and its sub-signature in which only 3 of the 17 genes contributed to the calculated score (LSC3) were reported to be highly prognostic in patients of diverse AML subtypes and AML patients with high LSC17/LSC3 scores had poor outcomes [35]. The analysis in database GSE76008 indicated that the expression of FBXO22 was positively correlated with LSC3 signature score, although its relationship with LSC17 could not be clearly shown (Additional file 6: Fig. S5B). In line with this, both mRNA and protein levels of FBXO22 in murine GFP^+^Lin^–^c-Kit^+^ enriched LSCs were elevated compared with normal Lin^–^c-Kit^+^ enriched HSCs (Additional file 6: Fig. S5C, D). Interestingly, the frequency of GFP^+^Mac-1^+^c-Kit^+^ (MK) enriched LSCs was reduced in primary *Fbxo22*^–/–^ transplanted mice compared with control mice (Fig. 4A), although there was no significant difference in survival time on primary transplantation of infected *Fbxo22*^–/–^ and *Fbxo22*^+/+^ cells (Additional file 4: Fig. S3E). We also measured frequency of GFP^+^Lin^−^CD127^−^c-Kit^+^CD16/32^+^CD34^+^ (LGMP) population, which has been suggested to be another more stringent way to determine LSCs [36], and demonstrated that the percentage of *Fbxo22*^–/–^ LGMP cells was also reduced in primary transplantation (Fig. 4B). As expected, the frequencies of LSCs were markedly reduced in the secondary *Fbxo22*^–/–^ transplanted mice compared with control mice (Fig. 4C, D). Similarly, a modestly and dramatically lower percentage of LSCs was detected in primary and secondary *Fbxo22*-null *Scl-Cre* transplanted mice model, respectively (Additional file 6: Fig. S5E–H). The reduction of LSCs frequency was also correlated with the reduced CFUs on first and secondary plating (Fig. 4E). Also, *Fbxo22* deletion significantly impaired the ability of LGMP to cause disease in secondary recipient mice (Fig. 4F). We performed a limiting dilution assay with sorted GFP^+^ *Fbxo22*^+/+^ and *Fbxo22*^–/–^ AML cells from primary recipients and found that the frequency of LSCs in *Fbxo22*^–/–^ AML mice (1:97,540 cells) was significantly lower compared with the *Fbxo22*^+/+^ mice (1:223 cells) (Fig. 4G). Furthermore, Hoechst and Ki67 staining revealed a significantly higher percentage of cycling cells (Hoechst^high^Ki67^high^) accompanied with a decrease in G_0_ quiescent cells (Hoechst^low^Ki67^low^) and G_1_ (Hoechst^low^Ki67 ^high^) cells in *Fbxo22*^–/–^ LGMPs (Fig. 4H). In addition, the proportion of annexin-V^+^ apoptotic cells was markedly greater within *Fbxo22*^–/–^ LGMPs (Fig. 4I). Similar results were observed in *Scl-Cre*;*Fbxo22*^*fl/fl*^ mice (Additional file 6: Fig. S5I-J). Collectively, *Fbxo22* deficiency in LSCs results in enhanced cell cycle entry and increased apoptosis.

### FBXO22 promotes degradation of BACH1 in MLLr AML cells

**Fig. 5:**
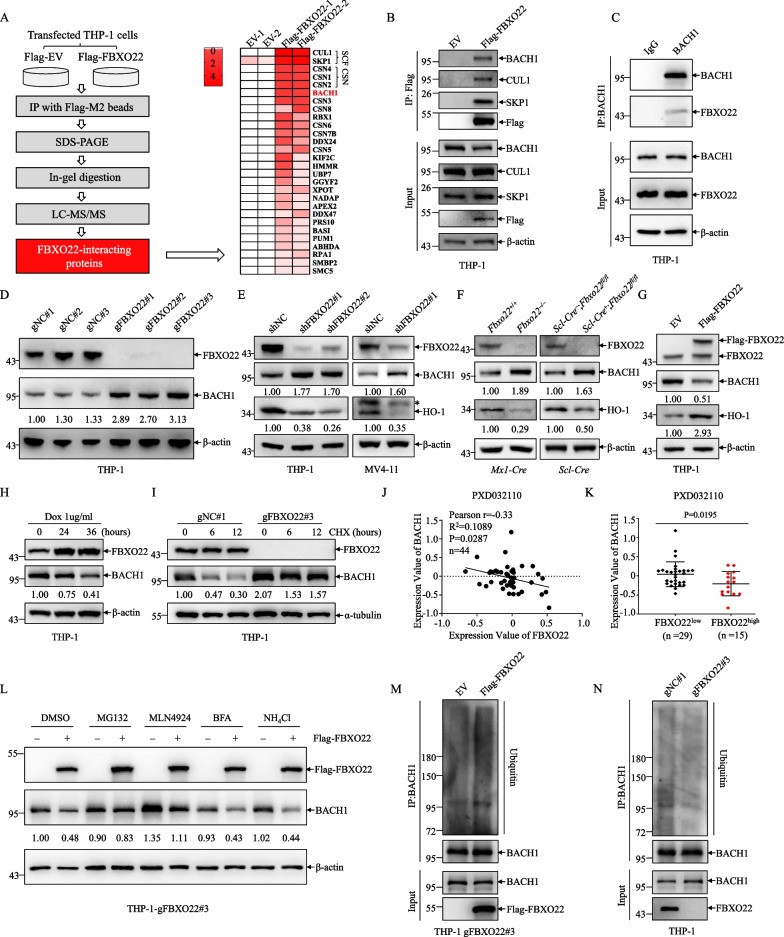
FBXO22 promotes degradation of BACH1 in MLLr AML cells. **A** The workflow for identifying FBXO22-interacting proteins in THP-1 cells by LC-MS/MS and the heat map analysis of 28 overlapping proteins identified in two independent experiments. **B** Western blot analysis of indicated proteins in the inputs and immunoprecipitates of Flag-tagged FBXO22 transfected THP-1 cells. **C** Western blot analysis of indicated proteins in the inputs and immunoprecipitates of endogenous BACH1 in THP-1 cells. **D** Western blot analysis of indicated proteins in three clonally derived THP-1 cell lines depleted of FBXO22 (gFBXO22#1, gFBXO22#2 and gFBXO22#3) with three negative controls (gNC). **E** Western blot analysis of indicated proteins in THP-1 and MV4-11 cells infected with shNC, shFBXO22#1 and/or shFBXO22#2. The asterisk represents a nonspecific band. **F** Western blot analysis of indicated proteins in MLL-AF9^+^ AML BM cells from primary recipients injected with *Fbxo22*^+/+^, *Fbxo22*^−/−^, *Scl-Cre*^+^;*Fbxo22*^*fl/fl*^ or *Scl-Cre*^−^;*Fbxo22*^*fl/fl*^ AML cells. **G** Western blot analysis of indicated proteins in THP-1 cells infected with EV and Flag-tagged FBXO22. **H** A Dox-inducible expression system encoding FBXO22 was introduced by lentiviruses into THP-1. Western blot analysis of indicated proteins in THP-1 cells by Dox administration for indicated times. **I** Western blot analysis of indicated proteins of THP-1 gNC#1 and gFBXO22#3 cell lines treated with CHX (100 μg/ml) for indicated times. **J** Pearson’s correlation between FBXO22 and BACH1 protein expression level of AML patients from PDX032110 database. **K** BACH1 protein expression level was compared between FBXO22^High^ and FBXO22^Low^ protein expression groups in PDX032110 database. Categorization of samples into FBXO22^High^ and FBXO22^Low^ protein expression was determined by maximally selected rank statistics method. **L** Western blot analysis of indicated proteins in THP-1 gFBXO22#3 cells infected with EV and Flag-tagged FBXO22 and treated with MG132 (10 μM), MLN4924 (10 μM), BFA (10 μM) and NH_4_Cl (10 mM) for 24 h. **M**, **N** THP-1 gFBXO22#3 cells infected with EV and Flag-tagged FBXO22 (**M**), THP-1 gNC#1 and gFBXO22#3 cells (**N**) were treated with 10 μM MG132 for 6 h, harvested, and submitted to in vivo ubiquitination assay, followed by Western blot analysis with indicated antibodies. Error bars denote mean ± SD. Statistical significance was determined by two-tailed unpaired *t* test (**K**) and the *P* values were shown. All experiments were repeated at least three times with similar results. The numbers in **D**–**I** and **L** show the quantification of protein levels

To investigate the molecular mechanisms underlying suppression of AML progression mediated by *Fbxo22* deficiency, we explored the interactome of FBXO22 in THP-1 cells by immunoprecipitation (IP) combined with LC-MS/MS analysis. In total, 28 overlapping candidates were identified in two independent experiments. In addition to two core subunits of SCF complex (SKP1 and CUL1) and three subunits of the COP9 signalosome complex (CSN), an essential regulator of the ubiquitin conjugation pathway [37], we noticed that BACH1, a regulator of oxidative stress response and a recently known substrate of FBXO22 [24], was reproducibly identified as the highest abundance of the FBXO22-interacting proteins in THP-1 cells (Fig. 5A, Additional file 7: Table S2), which could be confirmed by co-IP-based immunoblotting (Fig. 5B). The interaction between endogenous BACH1 and FBXO22 could be detected in THP-1 cells (Fig. 5C). Given that FBXO22 mediates the hemin-induced degradation of BACH1 in lung cancer cells [24], we assessed whether FBXO22 facilitates BACH1 degradation in MLLr AML cells. As shown in Fig. 5D, depletion of FBXO22 by CRISPR-CAS9 in THP-1 cells significantly increased the protein level of BACH1. Knockdown of FBXO22 by shRNA led to stabilization of BACH1 in THP-1 and MV4-11 cells, accompanied by reduction of heme oxygenase-1(HO-1), the best-known target repressor gene of BACH1 [38] (Fig. 5E). Meanwhile, the increased BACH1 and decreased HO-1 proteins could also be confirmed in *Fbxo22*-deficient GFP^+^ AML cells (Fig. 5F). In stark contrast, ectopic expression and inducible expression of FBXO22 strongly reduced BACH1 protein and/or increased HO-1 expression in THP-1 cells (Fig. 5G, H). Moreover, the decrease of BACH1 protein under cycloheximide (CHX) treatment was dramatically blocked in FBXO22 knockout THP-1 cells (Fig. 5I). Furthermore, we analyzed the correlation between FBXO22 and BACH1 protein expression in the proteomic database of AML (PXD032110) [39]. The results showed that the protein expression of FBXO22 was negatively correlated with BACH1 protein level in primary AML patients, and patients with higher FBXO22 protein expression had lower BACH1 protein expression and vice versa (Fig. 5J, K).

To validate whether FBXO22 degrades BACH1 through proteasome pathway, we transfected Flag-FBXO22 into FBXO22 knockout THP-1 cells and treated with proteasome inhibitor MG132 and CUL-RING ligase inhibitor MLN4924, along with lysosome inhibitors brefeldin A (BFA) and NH_4_Cl. The results showed that FBXO22 expression significantly decreased BACH1 protein, which was abundantly blocked by treatment with MG132 and MLN4924 rather than BFA and NH_4_Cl, suggesting that FBXO22-mediated BACH1 degradation is regulated by the proteasome and CRL complex (Fig. 5L). Moreover, FBXO22 knockout THP-1 cells were transfected with Flag-FBXO22 and empty vector and then subjected to IP with BACH1 antibody under denaturing conditions. Immunoblotting revealed that FBXO22 expression increased the abundance of ubiquitin conjugates observable on endogenous BACH1 purified protein (Fig. 5M). On the contrary, FBXO22 knockout reduced endogenous BACH1 ubiquitylation in THP-1 cells under denaturing conditions (Fig. 5N). These results indicate that FBXO22 interacts with BACH1 and promotes degradation of BACH1 in MLLr AML cells.

### BACH1 suppresses MLLr AML progression

Although some reports showed that *Bach1* and *Bach2* double-deficient mice repressed erythropoiesis and B-cell development, while support myelopoiesis in steady-state conditions [40], *Bach1* deficiency has also been reported to not apparently affect the repopulation ability of HSCs [41]. In addition, we introduced BACH1 in murine myeloid precursor 32D cells and Lin^–^ HSPC cells and found that BACH1 overexpression in normal HSCs had no effect on the cell proliferation and colony formation ability and did not change myeloid, lymphoid and erythroid lineage commitment (Additional file 8: Fig. S6A–E).

**Fig. 6:**
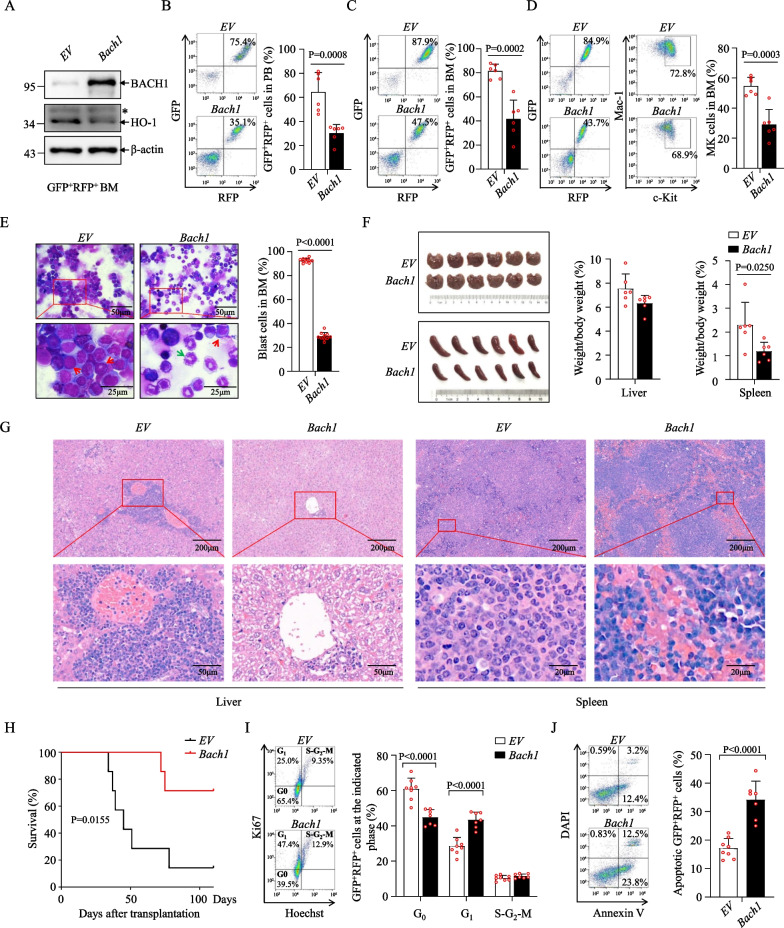
BACH1 suppresses MLLr AML progression. **A** Western blot analysis of indicated proteins in sorted GFP^+^RFP^+^ AML cells infected with *EV* and *Bach1*. The asterisk represents a nonspecific band. **B** Flow cytometry plots (left) and the percentage of GFP^+^RFP^+^ cells in PBMC (right) from the secondary recipients (*n* = 6). **C**, **D** Flow cytometry plots (left) and the percentage of GFP^+^RFP^+^ cells (**C**) or MKs (**D**) in BM from the secondary recipients (right, *n* = 6). **E** Representative images of Giemsa-Wright staining for *EV* and *Bach1* BM cells upon the second transplantation. Quantification of the frequencies of blast cells was shown on the right. Red and green arrows point to representative blast cells and differentiated cells, respectively. **F**, **G** Gross pathology (left) and relative weights (right) (F), hematoxylin–eosin staining (**G**) of the livers and spleens from the secondary recipients (*n* = 6). **H** Survival curves for recipients receiving *EV* and *Bach1* GFP^+^RFP^+^ cells upon secondary transplantation (*n* = 7). **I**, **J** Cell-cycle (**I**) or apoptosis (**J**) analysis of GFP^+^RFP^+^ cells in BM from the secondary recipients (*n* = 7–8). Error bars denote mean ± SD. Statistical significance was determined by two-tailed unpaired *t* test (**B**–**F**, and **I**, **J**) or log-rank test (**H**), and the *P* values were shown. All animal experiments were repeated at least twice with similar results

Several studies have established that BACH1 promotes progression of various types of cancers via multiple mechanisms, as reviewed [42]. Notably, BACH1 also functions as a tumor suppressor and controls growth and survival of AML cells by regulating HO-1 expression, suggesting that functional upregulation of BACH1 is a potential strategy for antileukemic therapy [43]. In line with this, BACH1 exhibited a significantly lower protein expression in murine GFP^+^Lin^–^c-Kit^+^ enriched LSCs compared with normal Lin^–^c-Kit^+^ enriched HSCs (Additional file 6: Fig. S5D). To investigate the functional role of elevated BACH1 expression in MLLr AML cells, we transfected Flag-BACH1 into MV4-11 cells and found that BACH1 overexpression significantly inhibited cell clonogenicity and increased cell apoptosis (Additional file 8: Fig. S6F–H). Furthermore, we introduced BACH1 by lentiviral BACH1-IRES-RFP plasmids in BM GFP^+^ cells collected from AML mice, followed by transplantation into irradiated recipient mice. Then, the GFP^+^RFP^+^ AML cells were sorted and injected into lethally irradiated mice (Additional file 8: Fig. S6I). Western blot analysis showed that BACH1 was highly expressed in GFP^+^RFP^+^ AML cells (Fig. 6A). Indeed, BACH1 overexpression obviously decreased the frequencies of GFP^+^RFP^+^ AML cells in PB and BM of recipient mice (Fig. 6B, C). More importantly, the percentage of LSCs and population of blast cells in BM under BACH1 overexpression were much lower than that in control group (Fig. 6D, E), together with decreased spleen size and weight (Fig. 6F), much less infiltration with leukemic cells in liver and spleen (Fig. 6G), and extended survival times in BACH1 overexpression recipient mice (Fig. 6H). Further mechanistic dissection revealed that BACH1 overexpression caused a higher percentage of cycling cells and a decrease in G_0_ quiescent cells (Fig. 6I). In addition, the proportion of annexin-V^+^ apoptotic cells was greatly enhanced in BACH1 overexpression recipient mice (Fig. 6J). Collectively, these data indicate that overexpression of BACH1 inhibits MLLr AML progression.

### *FBXO22*-mediated* BACH1* degradation enables AML progression

**Fig. 7:**
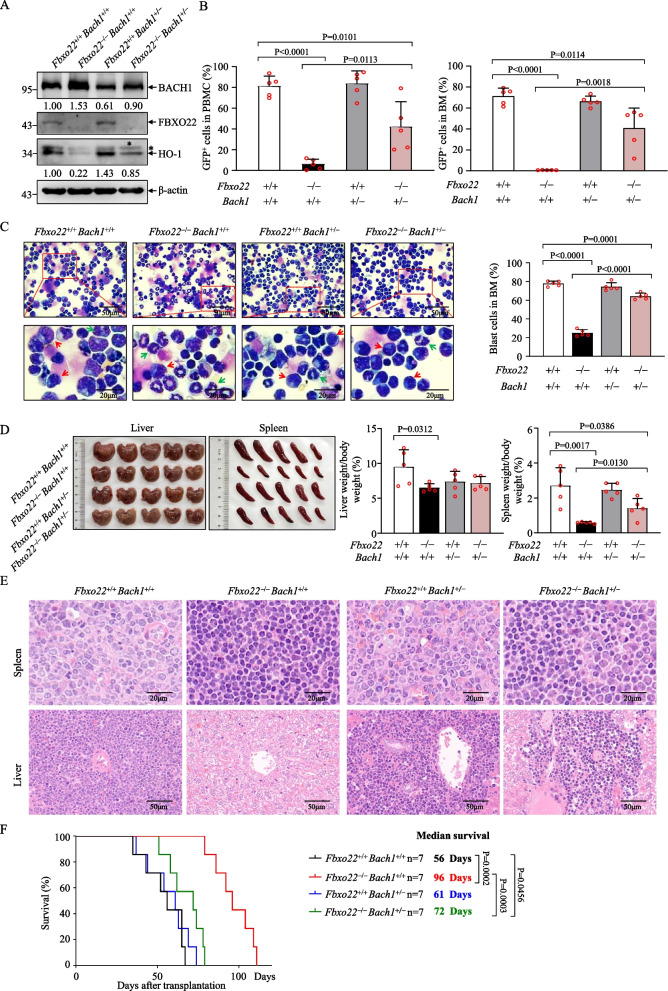
FBXO22-mediated degradation of BACH1 enables maintenance of AML progression. **A** The indicated proteins were evaluated by Western blot in BM GFP^+^ cells from the indicated mice upon primary transplantation. The numbers show the quantification of protein levels. The asterisk represents a nonspecific band. **B** The percentages of GFP^+^ cells in PBMC (left) or BM (right) from the secondary recipients transplanted with *Fbxo22*^+/+^*Bach1*^+/+^_,_ *Fbxo22*^−/−^*Bach1*^+/+^_,_ *Fbxo22*^+/+^*Bach1*^+/−^ and *Fbxo22*^−/−^*Bach1*^+/−^ AML cells (*n* = 5). **C** Representative images of Giemsa-Wright staining for BM cells from the indicated mice upon the second transplantation. Quantification of the frequencies of blast cells was shown on the right. **D**, **E** Gross pathology (left, **D**), relative weights (right, **D**) and hematoxylin–eosin staining (**E**) of the livers and spleens from the secondary recipients (*n* = 5). **F** Survival curves and analysis of median survival from secondary recipients injected with *Fbxo22*^+/+^*Bach1*^+/+^_,_ *Fbxo22*^−/−^*Bach1*^+/+^_,_ *Fbxo22*^+/+^*Bach1*^+/−^ and *Fbxo22*^−/−^*Bach1*^+/−^ AML cells (*n* = 7). Error bars denote mean ± SD. Statistical significance was determined by two-tailed unpaired *t* test (**B**–**D**) or log-rank test (**F**), and the *P* values were shown. All animal experiments were repeated at least twice with similar results

To detect whether BACH1 accumulation is involved in *Fbxo22* deficiency mediated inhibition of AML progression, we crossed *Mx1-Cre*;*Fbxo22*^*fl/fl*^ mice with *Bach*1^+/–^ mice to generate *Fbxo22*^+/+^*Bach1*^+/+^, *Fbxo22*^–/–^*Bach1*^+/+^, *Fbxo22*^+/+^*Bach1*^+/–^ and *Fbxo22*^–/–^*Bach1*^+/–^ mice in the presence of pIpC treatment. Then, these mice were used to induce AML by infection with MLL-AF9-GFP retrovirus. As depicted in Fig. 7A, *Bach1*^+/–^ mice had lower BACH1 and higher HO-1 expressions than *Bach1*^+/+^ mice, while efficient *Fbxo22* deletion induced higher BACH1 and lower HO-1 expressions in either *Fbxo22*^–/–^*Bach1*^+/+^ or *Fbxo22*^–/–^*Bach1*^+/–^ mice after primary transplantation, consistent with the notion that FBXO22 degraded BACH1 (Fig. 5D–F). Furthermore, we performed second transplantation with the same number of GFP^+^ AML cells from these mice. Although heterozygous *Bach1* loss in *Fbxo22*^+/+^ cells did not accelerate MLL-AF9-induced leukemogenesis, heterozygous *Bach1* loss in *Fbxo22*^–/–^ cells partially but significantly reversed delayed leukemogenesis in *Fbxo22*^–/–^ mice, as evidenced by percentages of GFP^+^ AML cells in peripheral blood mononuclear cell (PBMC), BM and blast cells in BM, sizes and weights of spleen/liver and tissue infiltration with leukemic cells (Fig. 7B–E). Accordingly, *Fbxo22*^–/–^*Bach1*^+/–^ mice presented poorer survival compared with *Fbxo22*^–/–^*Bach1*^+/+^ mice (Fig. 7F). Although it remains to be further investigated whether there is a dose-related effect of Bach1, all these data indicate that FBXO22-mediated degradation of BACH1 protein enables maintenance of AML progression.

## Discussion

In this report, we used two conditional knockout mouse models to study the role of FBXO22 in normal and malignant hematopoiesis. The *Mx1-Cre* has a very high recombination efficiency but is not hematopoietic cell-specific and also has a side effect from pIpC [31, 44]. The *Scl-Cre* is hematopoietic cell-specific but has incomplete gene deletion efficiency [32]. Although each model has its own advantage and disadvantage, the experimental results obtained from these models were consistent in both phenotype and functionality revealed by FBXO22 inactivation. Our results demonstrated that FBXO22 was required for MLLr AML development and maintenance of LSC function, but was dispensable for normal hematopoiesis at least during young mice. These findings provide a rationale for targeting FBXO22 to specifically eradicate AML LSCs but not their normal counterparts.

In contrast to FBXO9, whose expression is downregulated in AML patients compared to healthy controls and acting as a tumor suppressor in AML [17], FBXO22 is highly expressed in AML, especially MLLr AML patients. Therefore, we used MLL-AF9-induced mouse AML model to investigate the function of FBXO22 to AML development. Our observation demonstrated that deletion of FBXO22 significantly prevented MLL-AF9-induced AML leukemogenesis and reduced the number of LSCs in AML mice by inducing apoptosis, indicating that FBXO22 functions as an oncoprotein that critically involved in the development of AML. Interestingly, depletion of FBXO22 exhibited no significant effects on normal hematopoiesis in mice at the steady state except a slightly higher proportion of erythroid progenitor cells and weaker effect on HSC self-renewal under competitive repopulation. Notably, the increased CFU-E colonies upon FBXO22 deletion was more apparent than the higher TER119^+^ cells expression upon FBXO22 deletion, which might be due to the differences in in vitro and in vivo effects. Thus, FBXO22 appears to be uniquely hijacked to play an essential role in MLLr AML pathogenesis and LSC maintenance, as it is dispensable for normal hematopoiesis, suggesting that FBXO22 is a feasible target for AML therapy.

Consistent with the previous report [24], we demonstrated that FBXO22 promoted degradation of BACH1 in MLLr AML cells. Although FBXO22 was shown to interact with and induce degradation of BACH1 with ubiquitination, it remains to be further investigated whether FBXO22 is the direct E3 ligase of BACH1. The transcription factor BACH1, together with nuclear factor erythroid 2-related factor 2 (Nrf2) and the Maf transcription factors, controls the expression of HO-1 and other antioxidant genes [45, 46]. HO-1 is commonly regarded as a survival molecule, exerting an antiapoptotic role in cancer progression, and its inhibition is considered beneficial in a number of cancers [47, 48]. As a heme-regulated transcription factor, BACH1 represses iron- and heme-related genes in normal cells. Although several studies have revealed that BACH1 promotes invasion and metastasis in breast, lung, pancreatic and ovarian cancer by regulating various sets of genes beyond iron metabolism by activating the transcription of critical metastatic genes [24, 49–53], BACH1 may impose negative effects on cancer cell growth and survival by directly repressing a subset of proteasome genes in gallbladder cancer and cholangiocarcinoma cells [54, 55]. In addition, BACH1 also functions as a tumor suppressor and controls survival of AML cells by regulating HO-1 expression [43], suggesting that functional upregulation of BACH1 is an alternative therapeutic strategy for antileukemic therapy. As expected, our findings reveal that BACH1 overexpression suppresses AML progression and LSC maintenance through increasing apoptosis, consistent with the phenotype revealed by Fbxo22 deletion. Therefore, we hypothesize that the diverse roles of BACH1 are likely due to the selective regulation of target genes in different cellular and environmental contexts. Given that BACH1 promotes ferroptosis by repressing the expression of genes involved in glutathione (GSH) synthesis and iron handling [56], and ferroptosis as well as apoptosis have been shown to act as cancer suppressor mechanism [57], more efforts are required to validate whether BACH1-promoted ferroptosis is also involved in AML progression in the future.

Our further functional studies demonstrate that BACH1 is an essential target of FBXO22 in AML development. As critical downstream targets of BACH1, HO-1 level can also be indirectly regulated by FBXO22 in AML. Through this axis, increased expression of FBXO22 in AML patients promotes LSC maintenance, leading to poor prognosis. Of course, besides BACH1, other potential targets of FBXO22 identified herein may partially mediate the effects of FBXO22 in AML, which warrants further investigation. In conclusion, our results provide evidence that FBXO22 plays critical roles in AML development and LSC maintenance through degradation of critical target BACH1 but minimally affects normal hematopoiesis. Targeting FBXO22 signaling represents a very promising therapeutic strategy for the treatment of AML.

## Conclusions

Based on analysis on the mRNA expression levels of F-box proteins in multiple AML patient cohort, we found that FBXO22 is highly expressed in human AML, especially MLLr AML. Conditional deletion of Fbxo22 in HSC cells of mice significantly impairs MLL-AF9-induced mouse AML development and the function of LSCs during serial transplantation without significant influences on normal hematopoiesis. Mechanistically, FBXO22 triggers BACH1 degradation to promote MLLr AML progression. Therefore, targeting FBXO22 might be an ideal strategy to eradicate LSCs without influencing normal hematopoiesis.

## Supplementary Information

**Additional file 1**. **Table S1.** Reagents, primers and plasmids used and patients’ information

**Additional file 2**. **Figure S1.** FBXO22 is highly expressed in human AML and required for the growth of AML, especially MLLr AML cells. **A** Relative mRNA expression levels of F-box family members were analyzed in database GSE1159 (Normal n=5, AML n=285). Error bars denote mean ± SEM. **B** FBXO22 mRNA expression level in bone marrow or peripheral blood was analyzed in GSE7186 and GSE48173 databases. **C**, **D** AML 6# MNCs were infected with EV and Flag-tagged FBXO22, and FBXO22 protein was immunoblotted with β-actin as loading control (**C**). Cell numbers were counted at the indicated days (**D**). **E** Colony-forming assay (left) for AML 6# MNCs infected with EV and Flag-tagged FBXO22, and colony numbers at day 10 were counted (right, n=3). **F**, **G** FBXO22 mRNA expression level in bone marrow or peripheral blood from AML patients bearing various cytogenetic aberrations was analyzed in TCGA-LAML (**F**) or Beat-AML (**G**) database. **H** FBXO22 mRNA level in several MLLr and non-MLLr AML cell lines was evaluated by qRT-PCR. **I** FBXO22 protein in several MLLr and non-MLLr AML cell lines was immunoblotted with H3 as loading control. Error bars denote mean ± SD. Statistical significance was determined by two-tailed unpaired t test (**A**, **B** and **E**) , two-way ANOVA (**D**) or one-way ANOVA plus Fisher’s LSD test (**F**, **G**) and the P values were shown.

**Additional file 3**. **Figure S2. **Deletion of *Fbxo22* fails to affect normal hematopoiesis. **A** *Fbxo22* deletion was evaluated by genotyping in PBMCs from *Scl-Cre*^+^;*Fbxo22*^*fl/fl*^ and *Scl-Cre*^−^;*Fbxo22*^*fl/fl*^ mice at indicated times after tamoxifen treatment (left) and further evaluated by qRT-PCR (middle) or Western blot (right) in total BM cells at day 30 after tamoxifen treatment. **B**, **C** 32D cells infected with EV or HA-tagged Fbxo22 and indicated proteins were immunoblotted with β-actin as loading control (**B**), cell proliferation was examined by CCK-8 assay at the indicated days (**C**). **D** Colony-forming assay (left) for the indicated 32D cells, and colony numbers at day 7 after plating were counted (right, n=3). **E** Total number of BM cells was calculated in *Fbxo22*^+/+^ and *Fbxo22*^−/−^ mice (n=5). **F** Representative flow cytometry plots (left) and the percentage of CLPs (Lin^−^Sca-1^low^c-Kit^low^CD127^+^Flk2^+^) in BM from *Fbxo22*^+/+^ and *Fbxo22*^−/−^ mice (right, n=5). **G** Frequencies of myeloid (Mac-1^+^Gr-1^+^), erythroid (TER119^+^), B (B220^+^) and T (CD8^+^ or CD4^+^) cells in BM from *Fbxo22*^+/+^ and *Fbxo22*^−/−^ mice (n=5). **H** Flow cytometry plots (left) and apoptosis analysis (right) of LSK cells in BM from *Fbxo22*^+/+^ and *Fbxo22*^−/−^ (n=5-6). **I** Total BM cells from *Fbxo22*^+/+^ and *Fbxo22*^−/−^ mice were seeded in indicated methylcellulose medium for the quantification of CFU-GEMM, CFU-GM, CFU-pre-B and CFU-E colonies (n=3). **J**–**N** Total BM cells number (**J**) and percentages of indicated cells from BM (**K**–**N**) of *Scl-Cre*^+^;*Fbxo22*^*fl/fl*^ and *Scl-Cre*^−^;*Fbxo22*^*fl/fl*^ mice were analyzed (n=5). **O** Total BM cells from *Scl-Cre*^+^;*Fbxo22*^*fl/fl*^ and *Scl-Cre*^−^;*Fbxo22*^*fl/fl*^ mice were seeded in indicated methylcellulose medium for the quantification of CFU-GEMM, CFU-GM and CFU-E colonies (n=3). Error bars denote mean ± SD. Statistical significance was determined by two-tailed unpaired *t* test or two-way ANOVA (**C**) and the P values were shown. All animal experiments were repeated at least twice with similar results

**Additional file 4**. **Figure S3.** Loss of *Fbxo22* impairs MLL-AF9-induced AML development during serial transplantation. **A** Western blot analysis of indicated proteins in normal and MLL-AF9-IRES-GFP transfected murine BM cells. **B** Experimental strategy for generation of a mouse model of AML driven by MLL-AF9. **C**–**E** Percentages of GFP^+^ cells in PB (**C**) and GFP^+^ cells or MGs in BM (**D**) from the primary recipients and survival data (**E**) for these recipients transplanted with* Fbxo22*^+/+^ and *Fbxo22*^−/−^ AML cells were shown (n=5–6). **F**–**H** Percentages of GFP^+^ cells in PB (**F**) and GFP^+^ cells or MGs in BM (**G**) from the primary recipients and survival data (**H**) for these recipients transplanted with* Scl-Cre*^+^;*Fbxo22*^*fl/fl*^ and *Scl-Cre*^−^;*Fbxo22*^*fl/fl*^ AML cells were shown (n=4-6). Error bars denote mean ± SD. Statistical significance was determined by two-tailed unpaired *t* test (**C**, **D** and **F**, **G**) or log-rank test (**E** and **H**) and the P values were shown. All animal experiments were repeated at least twice with similar results

**Additional file 5**. **Figure S4.** Loss of *Fbxo22* impairs AML development during serial transplantation. **A**, **B** Percentages of GFP^+^ cells in PB (**A**), GFP^+^ cells and MGs in BM (**B**) from the *Scl-Cre*^+^;*Fbxo22*^*fl/fl*^ and *Scl-Cre*^−^;*Fbxo22*^*fl/fl*^ recipients upon the secondary transplantation (n=4). **C**, **D** Gross pathology (left) and relative weights (right, **C**), hematoxylin-eosin staining (**D**) of the livers and spleens from the secondary recipients (n=4). **E**, **F** Survival data for recipient mice receiving *Scl-Cre*^+^;*Fbxo22*^*fl/fl*^ and *Scl-Cre*^−^;*Fbxo22*^*fl/fl*^ AML cells upon the second (**E**), and third transplantation (**F**) (n=5). (G) GFP^+^ BM cells from the primary recipients transplanted with *Fbxo22*^+/+^ and *Fbxo22*^−/−^ AML cells injected into lethally irradiated recipients. Percentages of GFP^+^ cells in PB, spleen, and BM were analyzed at 16 h post-transplantation (n=6). **H**, **I** Secondary recipients transplanted with *Mx1-Cre*^+^;*Fbxo22*^*fl/fl*^ and *Mx1-Cre*^−^;*Fbxo22*^*fl/fl*^ AML cells were treated by pIpC at 7 days post-transplantation for 7 times to induce deletion of *Fbxo22.* *Fbxo22* deletion was evaluated by Western blot in BM cells from recipients after pIpC treatment (**H**). Survival curves for recipients were shown (**I**) (n=5-6). Error bars denote mean ± SD. Statistical significance was determined by two-tailed unpaired *t* test (**A**, **B**, **C** and **G**)  or log-rank test (**E**, **F** and **I**) and the P values were shown. All animal experiments were repeated at least twice similar results.

**Additional file 6**. **Figure S5.** Loss of *Fbxo22* impairs the function of LSCs. **A** FBXO22 mRNA expression level in HSC or LSC was analyzed in databases as indicated. In GSE63270, GSE138883 and GSE68172 datasets, HSCs were characterized, respectively, as Lin^–^CD34^+^CD38^−^CD90^+^CD45RA^+^, CD34^+^ and CD34^+^CD38^−^ cells from bone marrow of healthy donors. LSCs were defined as CD34^+^CD38^−^ in GSE63270 and CD34^+^ cells purified from bone marrow of AML patients in GSE138883 and GSE68172 datasets. **B** Pearson’s correlation between LSC17 (left) or LSC3 (right) signature score and FBXO22 mRNA expression level of AML patients from GSE76008 database. **C**, **D** FBXO22 expression level in Normal LKs or GFP^+^LKs was tested by qRT-PCR (**C**) or Western blot (**D**). **E**, **F** Flow cytometry plots (left) and the percentages of MKs (right, **E**) and LGMP (right, **F**) in BM from the primary recipients transplanted with *Scl-Cre*^+^;*Fbxo22*^*fl/fl*^ and *Scl-Cre*^−^;*Fbxo22*^*fl/fl*^ AML cells (n=6). **G**, **H** Flow cytometry plots (left) and the percentages of MKs (right, **G**) and LGMP (right, **H**) in BM from the secondary recipients transplanted with *Scl-Cre*^+^;*Fbxo22*^*fl/fl*^ and *Scl-Cre*^−^;*Fbxo22*^*fl/fl*^ AML cells (n=4). **I**, **J** Cell-cycle (**I**) or apoptosis (**J**) analysis of LGMP cells in BM from the secondary recipients transplanted with *Scl-Cre*^+^;*Fbxo22*^*fl/fl*^ and *Scl-Cre*^−^;*Fbxo22*^*fl/fl*^ AML cells (n=3-5). Error bars denote mean ± SD. Statistical significance was determined by two-tailed unpaired *t* test (**A**, **C** and **E**–**J**), and the P values were shown. All animal experiments were repeated at least twice with similar results

**Additional file 7**. **Table S2.** Mass spectrometry data

**Additional file 8**. **Figure S6.** BACH1 suppresses MLLr AML progression. **A**, **B** 32D cells infected with EV or Bach1 and indicated proteins were immunoblotted with β-actin as loading control (**A**), cell proliferation was examined by CCK-8 assay at the indicated days (**B**). **C** Colony-forming assay (left) for the indicated 32D cells, and colony numbers at day 7 after plating were counted (right, n=3). **D**, **E** Lin^−^ BM cells from wild-type mice infected with EV or Bach1 and indicated proteins were immunoblotted with β-actin as loading control (**D**), cells were seeded in indicated methylcellulose medium for the quantification of CFU-GEMM, CFU-GM, CFU-pre-B and CFU-E colonies (**E**, n=3). **F** MV4-11 cells were infected with Flag-tagged BACH1 or EV, indicated proteins were immunoblotted with β-actin as loading control. The asterisk represents a nonspecific band. **G** Representative images (left) of colonies and colony numbers (right, n=3) formed by MV4-11 cells infected with Flag-tagged BACH1 or EV. **H** Apoptosis analysis of MV4-11 cells infected with Flag-tagged BACH1 or EV. **I** Schematic strategy of evaluation of the in vivo effect of *Bach1 *overexpression in mouse AML cells. Error bars denote mean ± SD. Statistical significance was determined by two-tailed unpaired *t* test (**C**, **E** and **G**, **H**), two-way ANOVA (**B**) and the P values were shown. All experiments were repeated at least three times with similar results

**Additional file 9**. Uncropped Western blots

## Data Availability

The datasets used and/or analyzed during the current study are available from the GEO (https://www.ncbi.nlm.nih.gov/geo/), TCGA (https://cancergenome.nih.gov/), BeatAML2 (https://biodev.github.io/BeatAML2/) and Proteomic and Phosphoproteomic Landscapes of AML (http://www.leylab.org/amlproteome).

## Abbreviations

ACK: Ammonium chloride potassium
AML: Acute myeloid leukemia
APC: Allophycocyanin
ATCC: American Type Culture Collection
BACH1: BTB and CNC homology 1
BM: Bone marrow
BMMC: BM mononuclear cells
CCK 8: Cell counting kit 8
CDS: Coding sequence
CHX: Cycloheximide
CFU: Colony-forming unit
CFU-E: Colony-forming unit-erythrocyte
CFU-GEMM: Colony-forming unit-granulocyte, erythrocyte, monocyte and megakaryocyte
CFU-GM: Colony-forming unit-granulocyte and macrophage
CFU-Pre-B: Colony-forming unit-pre-B lymphocyte
CML: Chronic myeloid leukemia
CRLs: Cullin-RING ligases
CRUs: Competitive repopulating units
CSN: COP9 signalosome complex
CUL1: Cullin 1
DAPI: 4',6-diamidino-2-phenylindole
DMSO: Dimethyl sulfoxide
Dox: Doxycycline
FACS: Fluorescence Activating Cell Sorter
FBS: Fetal bovine serum
FBXL: F-box and leucine-rich repeat
FBXO: F-box only
FBXW: F-box and WD40 domain
GEO: Gene expression omnibus
GSH: Glutathione
HO-1: Heme oxygenase-1
HRP: Horseradish peroxidase
HSPCs: Hematopoietic stem/progenitor cells
H3: Histone 3
IL-3: Interleukin-3
IL-6: Interleukin-6
IP: Immunoprecipitation
KDM: Lysine demethylase
LC-MS/MS: Liquid chromatography-tandem mass spectrometry
LKB1: Liver kinase B
LGMP: Leukemic-granulocyte monocyte progenitor
LSCs: Leukemia stem cells
LSC3: A 3-gene LSC score
LSC17: A 17-gene LSC score
LT: Long-term
MK: Mac-1^+^c-Kit^+^
MLL: Mixed lineage leukemia
MM: Multiple myeloma
MNCs: Mononuclear cells
*Mx1*: Myxovirus resistance protein 1
NC: Normal control
Nrf2: Nuclear factor erythroid 2-related factor 2
PB: Peripheral blood
PBMC: Peripheral blood mononuclear cell
PBS: Phosphate buffer saline
PEI: Polyethylenimine
pIpC: Polyinosinic–polycytidylic acid
PTEN: Phosphatase and tension homologue
RBX1: RING-box protein 1
SCF: SKP1-CUL1-F-box
*Scl*: Stem cell leukemia stem-cell enhancer
SDS-PAGE: Sodium dodecyl sulfate polyacrylamide gel electrophoresis
SKP1: S-phase kinase-associated protein 1
T-ALL: T-cell acute lymphoblastic leukemia
TCGA: The Cancer Genome Atlas
TMT: Tandem mass tag
5-FU: 5-fluorouracil

## References

[1] Dohner H, Weisdorf DJ, Bloomfield CD (2015). Acute myeloid leukemia. N Engl J Med.

[2] Ley TJ, Miller C, Ding L, Raphael BJ, Mungall AJ, Cancer Genome Atlas Research N (2013). Genomic and epigenomic landscapes of adult de novo acute myeloid leukemia. N Engl J Med.

[3] Shlush LI, Mitchell A, Heisler L, Abelson S, Ng SWK, Trotman-Grant A (2017). Tracing the origins of relapse in acute myeloid leukaemia to stem cells. Nature.

[4] Shlush LI, Zandi S, Mitchell A, Chen WC, Brandwein JM, Gupta V (2014). Identification of pre-leukaemic haematopoietic stem cells in acute leukaemia. Nature.

[5] Muntean AG, Hess JL (2012). The pathogenesis of mixed-lineage leukemia. Annu Rev Pathol.

[6] Meyer C, Hofmann J, Burmeister T, Groger D, Park TS, Emerenciano M (2013). The MLL recombinome of acute leukemias in 2013. Leukemia.

[7] Dombret H, Gardin C (2016). An update of current treatments for adult acute myeloid leukemia. Blood.

[8] Dohner H, Estey E, Grimwade D, Amadori S, Appelbaum FR, Buchner T (2017). Diagnosis and management of AML in adults: 2017 ELN recommendations from an international expert panel. Blood.

[9] Skaar JR, Pagan JK, Pagano M (2014). SCF ubiquitin ligase-targeted therapies. Nat Rev Drug Discov.

[10] Wang Z, Liu P, Inuzuka H, Wei W (2014). Roles of F-box proteins in cancer. Nat Rev Cancer.

[11] Thompson BJ, Buonamici S, Sulis ML, Palomero T, Vilimas T, Basso G (2007). The SCFFBW7 ubiquitin ligase complex as a tumor suppressor in T cell leukemia. J Exp Med.

[12] Takeishi S, Matsumoto A, Onoyama I, Naka K, Hirao A, Nakayama KI (2013). Ablation of Fbxw7 eliminates leukemia-initiating cells by preventing quiescence. Cancer Cell.

[13] Chen BB, Glasser JR, Coon TA, Zou C, Miller HL, Fenton M (2012). F-box protein FBXL2 targets cyclin D2 for ubiquitination and degradation to inhibit leukemic cell proliferation. Blood.

[14] Ueda T, Nagamachi A, Takubo K, Yamasaki N, Matsui H, Kanai A (2015). Fbxl10 overexpression in murine hematopoietic stem cells induces leukemia involving metabolic activation and upregulation of Nsg2. Blood.

[15] Wang L, Feng W, Yang X, Yang F, Wang R, Ren Q (2018). Fbxw11 promotes the proliferation of lymphocytic leukemia cells through the concomitant activation of NF-kappaB and beta-catenin/TCF signaling pathways. Cell Death Dis.

[16] Fernandez-Saiz V, Targosz BS, Lemeer S, Eichner R, Langer C, Bullinger L (2013). SCFFbxo9 and CK2 direct the cellular response to growth factor withdrawal via Tel2/Tti1 degradation and promote survival in multiple myeloma. Nat Cell Biol.

[17] Hynes-Smith RW, Swenson SA, Vahle H, Wittorf KJ, Caplan M, Amador C (2019). Loss of FBXO9 enhances proteasome activity and promotes aggressiveness in acute myeloid leukemia. Cancers (Basel).

[18] Han Q, Zhang Q, Song H, Bamme Y, Song C, Ge Z (2020). FBXW4 Is highly expressed and associated with poor survival in acute myeloid leukemia. Front Oncol.

[19] Cheng J, Lin M, Chu M, Gong L, Bi Y, Zhao Y (2020). Emerging role of FBXO22 in carcinogenesis. Cell Death Discov.

[20] Ge MK, Zhang N, Xia L, Zhang C, Dong SS, Li ZM (2020). FBXO22 degrades nuclear PTEN to promote tumorigenesis. Nat Commun.

[21] Tian X, Dai S, Sun J, Jin G, Jiang S, Meng F (2015). F-box protein FBXO22 mediates polyubiquitination and degradation of KLF4 to promote hepatocellular carcinoma progression. Oncotarget.

[22] Zhu XN, He P, Zhang L, Yang S, Zhang HL, Zhu D (2019). FBXO22 mediates polyubiquitination and inactivation of LKB1 to promote lung cancer cell growth. Cell Death Dis.

[23] Sun R, Xie HY, Qian JX, Huang YN, Yang F, Zhang FL (2018). FBXO22 possesses both protumorigenic and antimetastatic roles in breast cancer progression. Cancer Res.

[24] Lignitto L, LeBoeuf SE, Homer H, Jiang S, Askenazi M, Karakousi TR (2019). Nrf2 activation promotes lung cancer metastasis by inhibiting the degradation of Bach1. Cell.

[25] Johmura Y, Sun J, Kitagawa K, Nakanishi K, Kuno T, Naiki-Ito A (2016). SCF(Fbxo22)-KDM4A targets methylated p53 for degradation and regulates senescence. Nat Commun.

[26] Johmura Y, Maeda I, Suzuki N, Wu W, Goda A, Morita M (2018). Fbxo22-mediated KDM4B degradation determines selective estrogen receptor modulator activity in breast cancer. J Clin Invest.

[27] Bai J, Wu K, Cao MH, Yang Y, Pan Y, Liu H (2019). SCF(FBXO22) targets HDM2 for degradation and modulates breast cancer cell invasion and metastasis. Proc Natl Acad Sci U S A.

[28] Yang S, Zhu XN, Zhang HL, Yang Q, Wei YS, Zhu D (2021). ANP32B-mediated repression of p53 contributes to maintenance of normal and CML stem cells. Blood.

[29] Shen SM, Zhang C, Ge MK, Dong SS, Xia L, He P (2019). PTENalpha and PTENbeta promote carcinogenesis through WDR5 and H3K4 trimethylation. Nat Cell Biol.

[30] Bottomly D, Long N, Schultz AR, Kurtz SE, Tognon CE, Johnson K (2022). Integrative analysis of drug response and clinical outcome in acute myeloid leukemia. Cancer Cell.

[31] Velasco-Hernandez T, Sawen P, Bryder D, Cammenga J (2016). Potential pitfalls of the Mx1-Cre system: implications for experimental modeling of normal and malignant hematopoiesis. Stem Cell Rep.

[32] Gothert JR, Gustin SE, Hall MA, Green AR, Gottgens B, Izon DJ (2005). In vivo fate-tracing studies using the Scl stem cell enhancer: embryonic hematopoietic stem cells significantly contribute to adult hematopoiesis. Blood.

[33] Krivtsov AV, Armstrong SA (2007). MLL translocations, histone modifications and leukaemia stem-cell development. Nat Rev Cancer.

[34] Thomas D, Majeti R (2017). Biology and relevance of human acute myeloid leukemia stem cells. Blood.

[35] Ng SW, Mitchell A, Kennedy JA, Chen WC, McLeod J, Ibrahimova N (2016). A 17-gene stemness score for rapid determination of risk in acute leukaemia. Nature.

[36] Krivtsov AV, Twomey D, Feng Z, Stubbs MC, Wang Y, Faber J (2006). Transformation from committed progenitor to leukaemia stem cell initiated by MLL-AF9. Nature.

[37] Dubiel W, Chaithongyot S, Dubiel D, Naumann M (2020). The COP9 signalosome: a multi-DUB complex. Biomolecules.

[38] Suzuki H, Tashiro S, Sun J, Doi H, Satomi S, Igarashi K (2003). Cadmium induces nuclear export of Bach1, a transcriptional repressor of heme oxygenase-1 gene. J Biol Chem.

[39] Kramer MH, Zhang Q, Sprung R, Day RB, Erdmann-Gilmore P, Li Y (2022). Proteomic and phosphoproteomic landscapes of acute myeloid leukemia. Blood.

[40] Kato H, Itoh-Nakadai A, Matsumoto M, Ishii Y, Watanabe-Matsui M, Ikeda M (2018). Infection perturbs Bach2- and Bach1-dependent erythroid lineage 'choice' to cause anemia. Nat Immunol.

[41] Ota K, Brydun A, Itoh-Nakadai A, Sun J, Igarashi K (2014). Bach1 deficiency and accompanying overexpression of heme oxygenase-1 do not influence aging or tumorigenesis in mice. Oxid Med Cell Longev.

[42] Zhang X, Guo J, Wei X, Niu C, Jia M, Li Q (2018). Bach1: function, regulation, and involvement in disease. Oxid Med Cell Longev.

[43] Miyazaki T, Kirino Y, Takeno M, Samukawa S, Hama M, Tanaka M (2010). Expression of heme oxygenase-1 in human leukemic cells and its regulation by transcriptional repressor Bach1. Cancer Sci.

[44] Essers MA, Offner S, Blanco-Bose WE, Waibler Z, Kalinke U, Duchosal MA (2009). IFNalpha activates dormant haematopoietic stem cells in vivo. Nature.

[45] Sun J, Brand M, Zenke Y, Tashiro S, Groudine M, Igarashi K (2004). Heme regulates the dynamic exchange of Bach1 and NF-E2-related factors in the Maf transcription factor network. Proc Natl Acad Sci U S A.

[46] Warnatz HJ, Schmidt D, Manke T, Piccini I, Sultan M, Borodina T (2011). The BTB and CNC homology 1 (BACH1) target genes are involved in the oxidative stress response and in control of the cell cycle. J Biol Chem.

[47] Loboda A, Jozkowicz A, Dulak J (2015). HO-1/CO system in tumor growth, angiogenesis and metabolism - Targeting HO-1 as an anti-tumor therapy. Vascul Pharmacol.

[48] Nitti M, Piras S, Marinari UM, Moretta L, Pronzato MA, Furfaro AL (2017). HO-1 induction in cancer progression: a matter of cell adaptation. Antioxidants (Basel).

[49] Liang Y, Wu H, Lei R, Chong RA, Wei Y, Lu X (2012). Transcriptional network analysis identifies BACH1 as a master regulator of breast cancer bone metastasis. J Biol Chem.

[50] Yang HL, Kuo YH, Tsai CT, Huang YT, Chen SC, Chang HW (2011). Anti-metastatic activities of Antrodia camphorata against human breast cancer cells mediated through suppression of the MAPK signaling pathway. Food Chem Toxicol.

[51] Wiel C, Le Gal K, Ibrahim MX, Jahangir CA, Kashif M, Yao H (2019). BACH1 stabilization by antioxidants stimulates lung cancer metastasis. Cell.

[52] Sato M, Matsumoto M, Saiki Y, Alam M, Nishizawa H, Rokugo M (2020). BACH1 promotes pancreatic cancer metastasis by repressing epithelial genes and enhancing epithelial-mesenchymal transition. Cancer Res.

[53] Han W, Zhang Y, Niu C, Guo J, Li J, Wei X (2019). BTB and CNC homology 1 (Bach1) promotes human ovarian cancer cell metastasis by HMGA2-mediated epithelial-mesenchymal transition. Cancer Lett.

[54] Jiang TY, Pan YF, Wan ZH, Lin YK, Zhu B, Yuan ZG (2020). PTEN status determines chemosensitivity to proteasome inhibition in cholangiocarcinoma. Sci Transl Med.

[55] Jiang TY, Feng XF, Fang Z, Cui XW, Lin YK, Pan YF (2021). PTEN deficiency facilitates the therapeutic vulnerability to proteasome inhibitor bortezomib in gallbladder cancer. Cancer Lett.

[56] Nishizawa H, Matsumoto M, Shindo T, Saigusa D, Kato H, Suzuki K (2020). Ferroptosis is controlled by the coordinated transcriptional regulation of glutathione and labile iron metabolism by the transcription factor BACH1. J Biol Chem.

[57] Jiang L, Kon N, Li T, Wang SJ, Su T, Hibshoosh H (2015). Ferroptosis as a p53-mediated activity during tumour suppression. Nature.

