# Supplementary figures and images for: FBXO22 promotes leukemogenesis by targeting BACH1 in MLL-rearranged acute myeloid leukemia
Source: J Hematol Oncol. 2023 Feb 11;16:9. doi: 10.1186/s13045-023-01400-0 (PMC9922468; doi:10.1186/s13045-023-01400-0)

Supplementary Figure S1

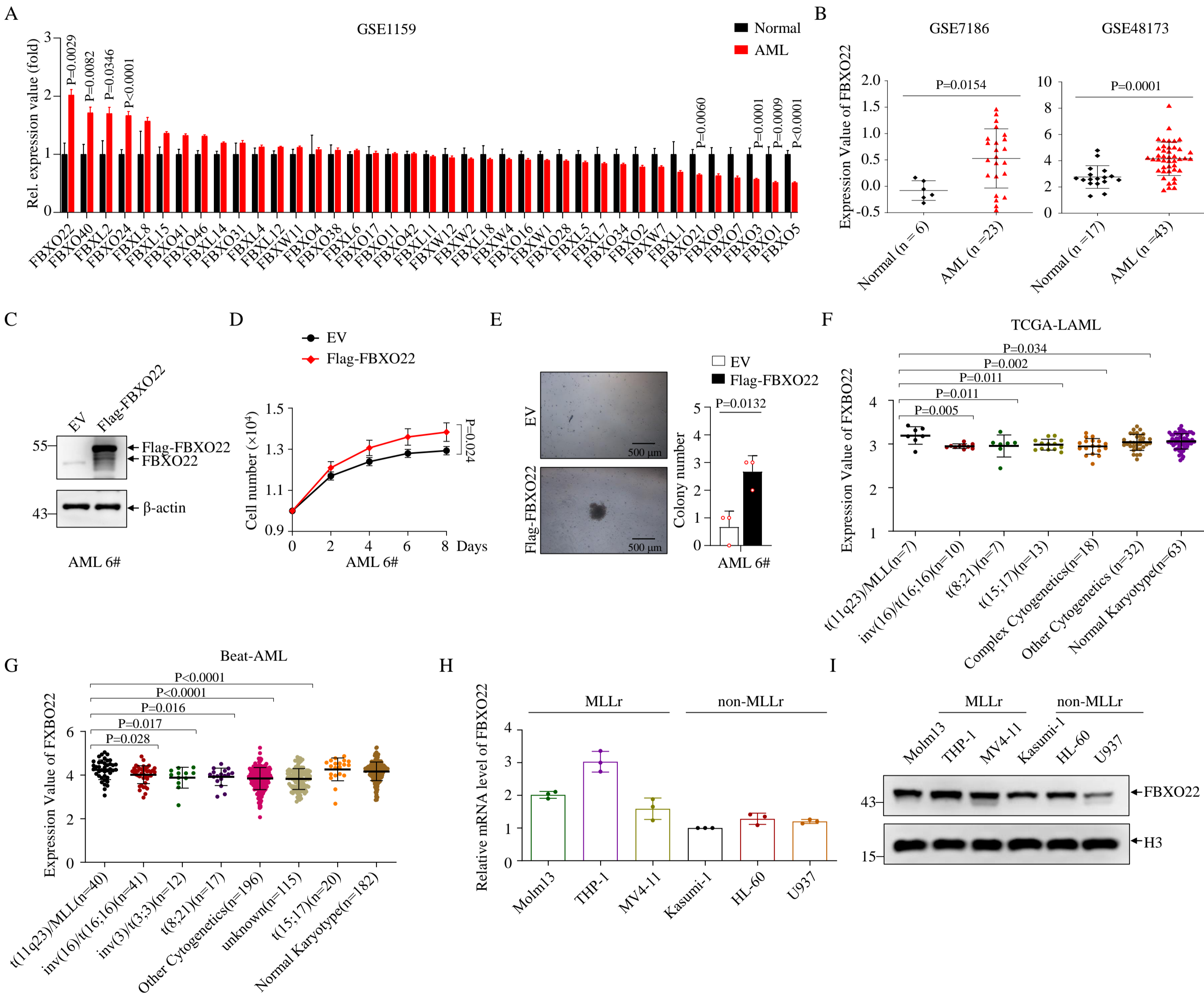

Supplement: Supplementary file 2 — Additional file 2. Figure S1. FBXO22 is highly expressed in human AML and required for the growth of AML, especially MLLr AML cells. A Relative mRNA expression levels of F-box family members were analyzed in database GSE1159 (Normal n=5, AML n=285). Error bars denote mean ± SEM. B FBXO22 mRNA expression level in bone marrow or peripheral blood was analyzed in GSE7186 and GSE48173 databases. C, D AML 6# MNCs were infected with EV and Flag-tagged FBXO22, and FBXO22 protein was immunoblotted with β-actin as loading control (C). Cell numbers were counted at the indicated days (D). E Colony-forming assay (left) for AML 6# MNCs infected with EV and Flag-tagged FBXO22, and colony numbers at day 10 were counted (right, n=3). F, G FBXO22 mRNA expression level in bone marrow or peripheral blood from AML patients bearing various cytogenetic aberrations was analyzed in TCGA-LAML (F) or Beat-AML (G) database. H FBXO22 mRNA level in several MLLr and non-MLLr AML cell lines was evaluated by qRT-PCR. I FBXO22 protein in several MLLr and non-MLLr AML cell lines was immunoblotted with H3 as loading control. Error bars denote mean ± SD. Statistical significance was determined by two-tailed unpaired t test (A, B and E) , two-way ANOVA (D) or one-way ANOVA plus Fisher’s LSD test (F, G) and the P values were shown. [file 13045_2023_1400_MOESM2_ESM.pdf]

## Supplementary Figure S2

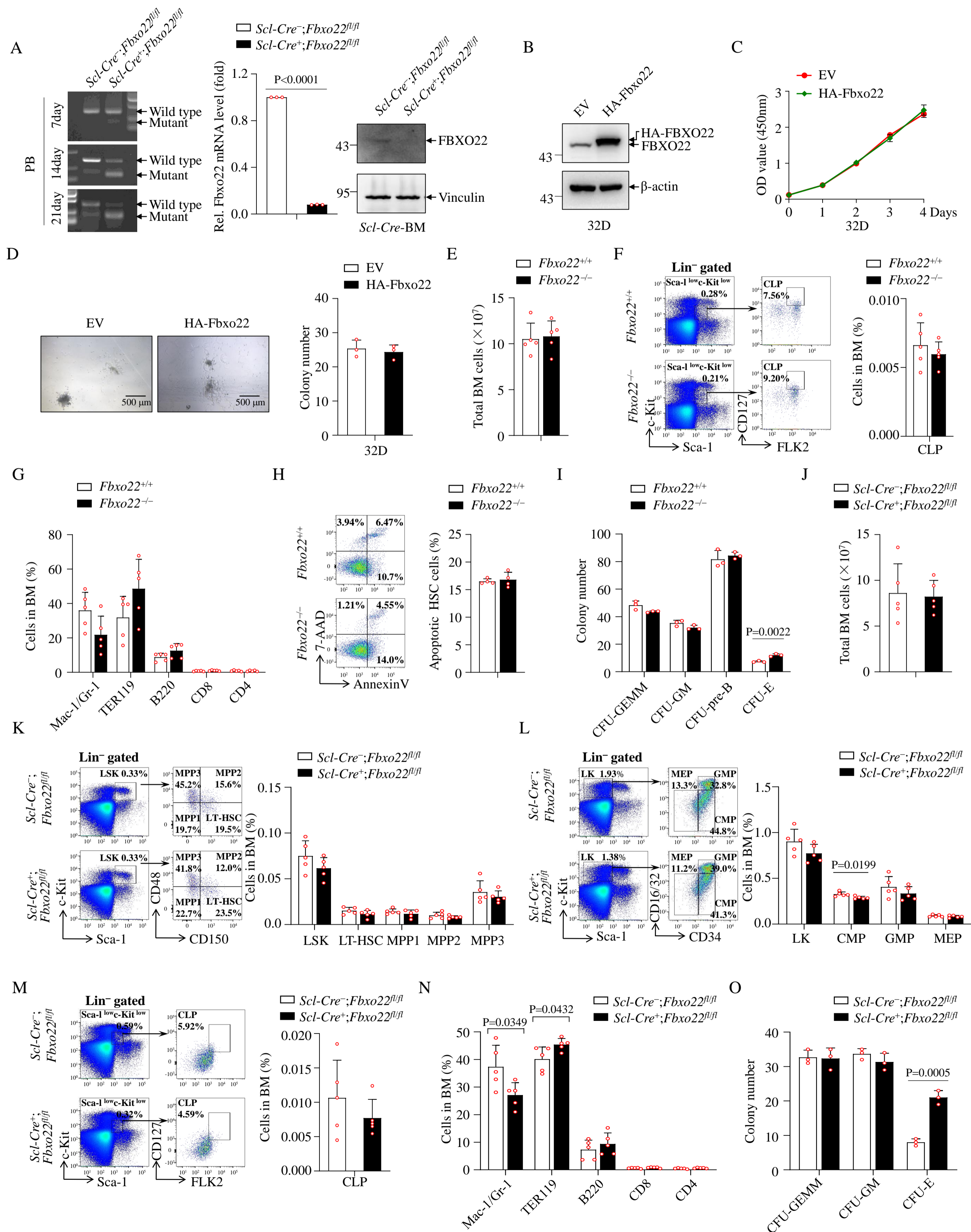

Supplement: Supplementary file 3 — Additional file 3. Figure S2. Deletion of Fbxo22 fails to affect normal hematopoiesis. A Fbxo22 deletion was evaluated by genotyping in PBMCs from Scl-Cre+;Fbxo22fl/fl and Scl-Cre−;Fbxo22fl/fl mice at indicated times after tamoxifen treatment (left) and further evaluated by qRT-PCR (middle) or Western blot (right) in total BM cells at day 30 after tamoxifen treatment. B, C 32D cells infected with EV or HA-tagged Fbxo22 and indicated proteins were immunoblotted with β-actin as loading control (B), cell proliferation was examined by CCK-8 assay at the indicated days (C). D Colony-forming assay (left) for the indicated 32D cells, and colony numbers at day 7 after plating were counted (right, n=3). E Total number of BM cells was calculated in Fbxo22+/+ and Fbxo22−/− mice (n=5). F Representative flow cytometry plots (left) and the percentage of CLPs (Lin−Sca-1lowc-KitlowCD127+Flk2+) in BM from Fbxo22+/+ and Fbxo22−/− mice (right, n=5). G Frequencies of myeloid (Mac-1+Gr-1+), erythroid (TER119+), B (B220+) and T (CD8+ or CD4+) cells in BM from Fbxo22+/+ and Fbxo22−/− mice (n=5). H Flow cytometry plots (left) and apoptosis analysis (right) of LSK cells in BM from Fbxo22+/+ and Fbxo22−/− (n=5-6). I Total BM cells from Fbxo22+/+ and Fbxo22−/− mice were seeded in indicated methylcellulose medium for the quantification of CFU-GEMM, CFU-GM, CFU-pre-B and CFU-E colonies (n=3). J–N Total BM cells number (J) and percentages of indicated cells from BM (K–N) of Scl-Cre+;Fbxo22fl/fl and Scl-Cre−;Fbxo22fl/fl mice were analyzed (n=5). O Total BM cells from Scl-Cre+;Fbxo22fl/fl and Scl-Cre−;Fbxo22fl/fl mice were seeded in indicated methylcellulose medium for the quantification of CFU-GEMM, CFU-GM and CFU-E colonies (n=3). Error bars denote mean ± SD. Statistical significance was determined by two-tailed unpaired t test or two-way ANOVA (C) and the P values were shown. All animal experiments were repeated at least twice with similar results [file 13045_2023_1400_MOESM3_ESM.pdf]

## Supplementary Figure S3

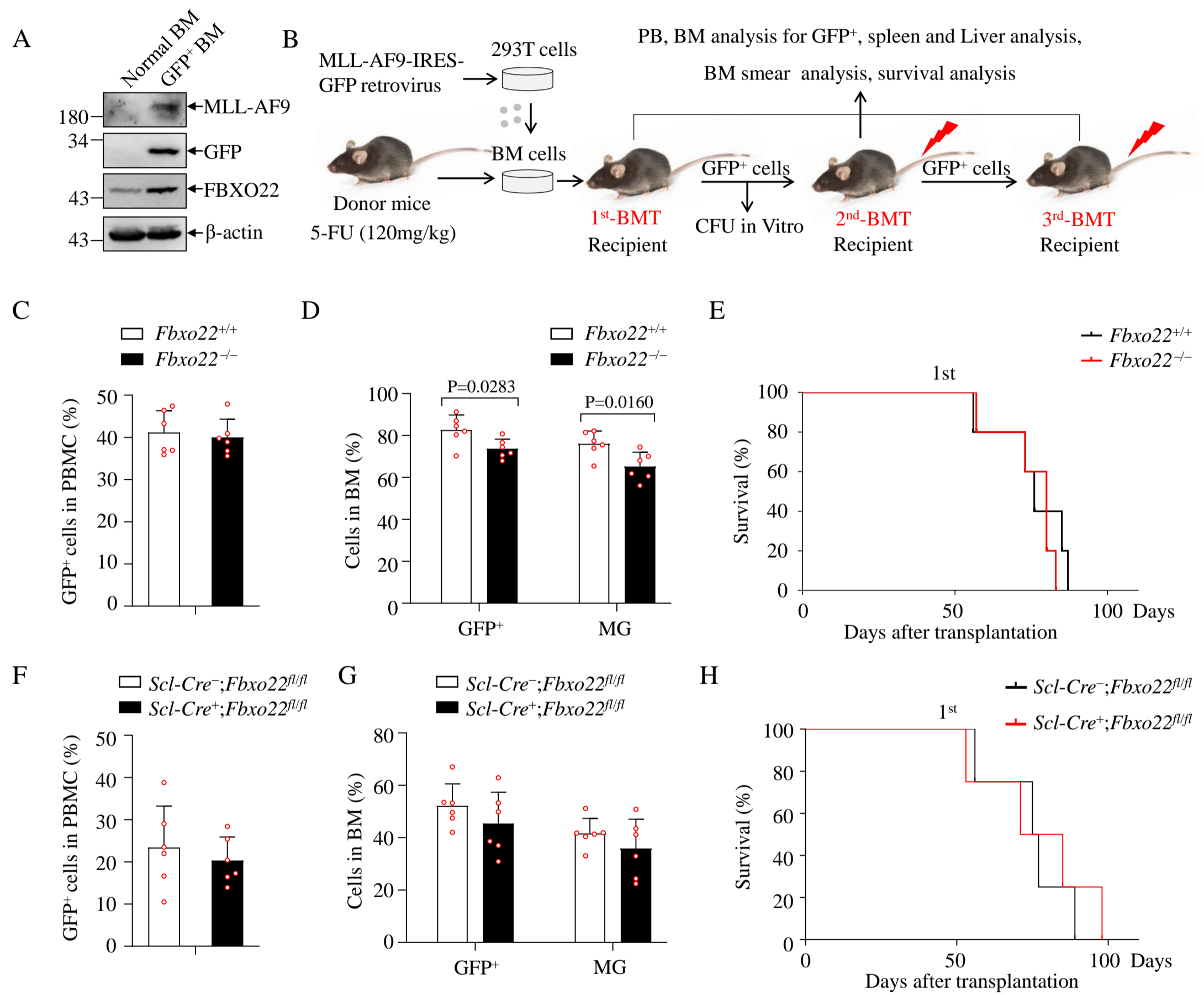

Supplement: Supplementary file 4 — Additional file 4. Figure S3. Loss of Fbxo22 impairs MLL-AF9-induced AML development during serial transplantation. A Western blot analysis of indicated proteins in normal and MLL-AF9-IRES-GFP transfected murine BM cells. B Experimental strategy for generation of a mouse model of AML driven by MLL-AF9. C–E Percentages of GFP+ cells in PB (C) and GFP+ cells or MGs in BM (D) from the primary recipients and survival data (E) for these recipients transplanted with Fbxo22+/+ and Fbxo22−/− AML cells were shown (n=5–6). F–H Percentages of GFP+ cells in PB (F) and GFP+ cells or MGs in BM (G) from the primary recipients and survival data (H) for these recipients transplanted with Scl-Cre+;Fbxo22fl/fl and Scl-Cre−;Fbxo22fl/fl AML cells were shown (n=4-6). Error bars denote mean ± SD. Statistical significance was determined by two-tailed unpaired t test (C, D and F, G) or log-rank test (E and H) and the P values were shown. All animal experiments were repeated at least twice with similar results [file 13045_2023_1400_MOESM4_ESM.pdf]

Supplementary Figure S4

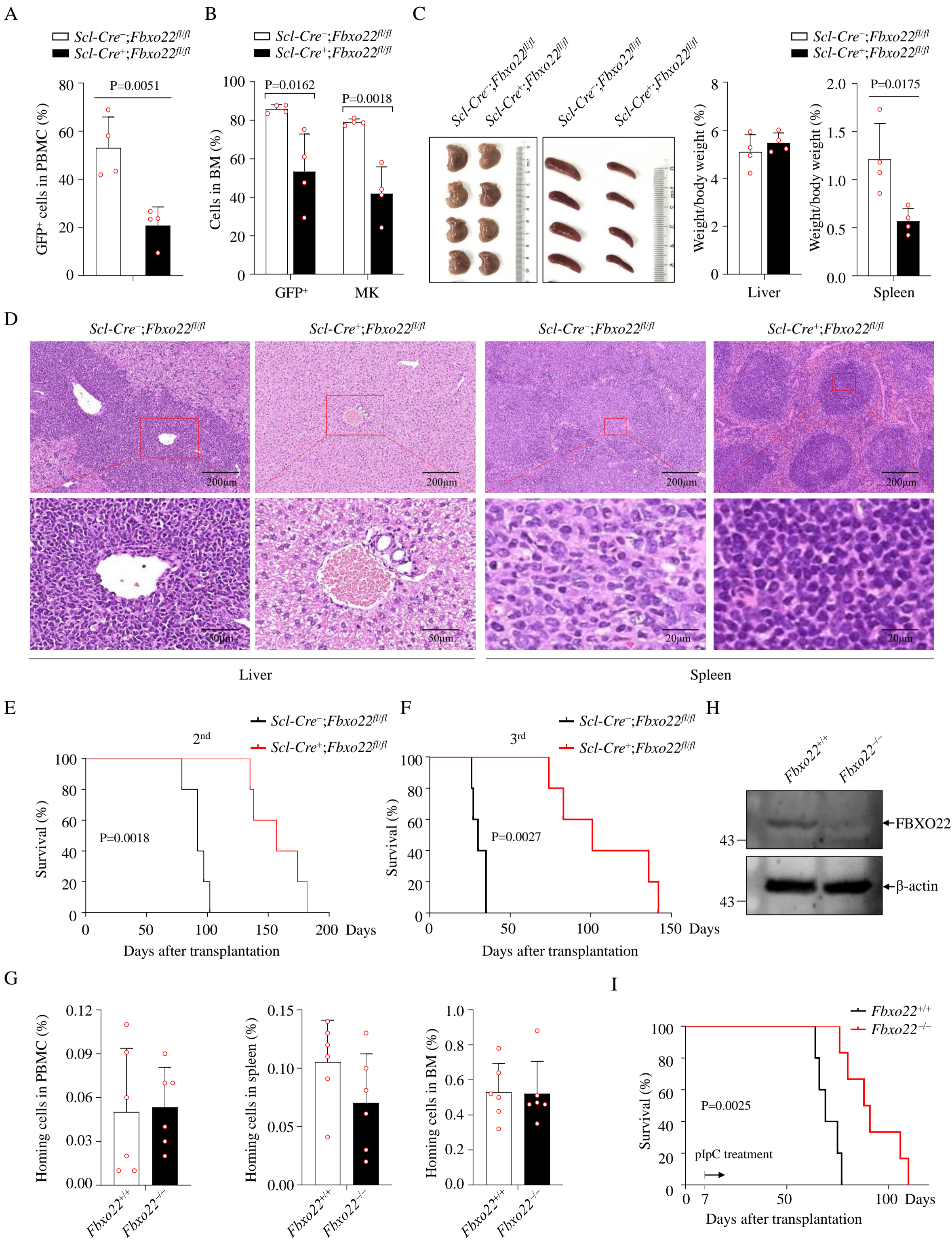

Supplement: Supplementary file 5 — Additional file 5. Figure S4. Loss of Fbxo22 impairs AML development during serial transplantation. A, B Percentages of GFP+ cells in PB (A), GFP+ cells and MGs in BM (B) from the Scl-Cre+;Fbxo22fl/fl and Scl-Cre−;Fbxo22fl/fl recipients upon the secondary transplantation (n=4). C, D Gross pathology (left) and relative weights (right, C), hematoxylin-eosin staining (D) of the livers and spleens from the secondary recipients (n=4). E, F Survival data for recipient mice receiving Scl-Cre+;Fbxo22fl/fl and Scl-Cre−;Fbxo22fl/fl AML cells upon the second (E), and third transplantation (F) (n=5). (G) GFP+ BM cells from the primary recipients transplanted with Fbxo22+/+ and Fbxo22−/− AML cells injected into lethally irradiated recipients. Percentages of GFP+ cells in PB, spleen, and BM were analyzed at 16 h post-transplantation (n=6). H, I Secondary recipients transplanted with Mx1-Cre+;Fbxo22fl/fl and Mx1-Cre−;Fbxo22fl/fl AML cells were treated by pIpC at 7 days post-transplantation for 7 times to induce deletion of Fbxo22. Fbxo22 deletion was evaluated by Western blot in BM cells from recipients after pIpC treatment (H). Survival curves for recipients were shown (I) (n=5-6). Error bars denote mean ± SD. Statistical significance was determined by two-tailed unpaired t test (A, B, C and G) or log-rank test (E, F and I) and the P values were shown. All animal experiments were repeated at least twice similar results. [file 13045_2023_1400_MOESM5_ESM.pdf]

# Supplementary Figure S5

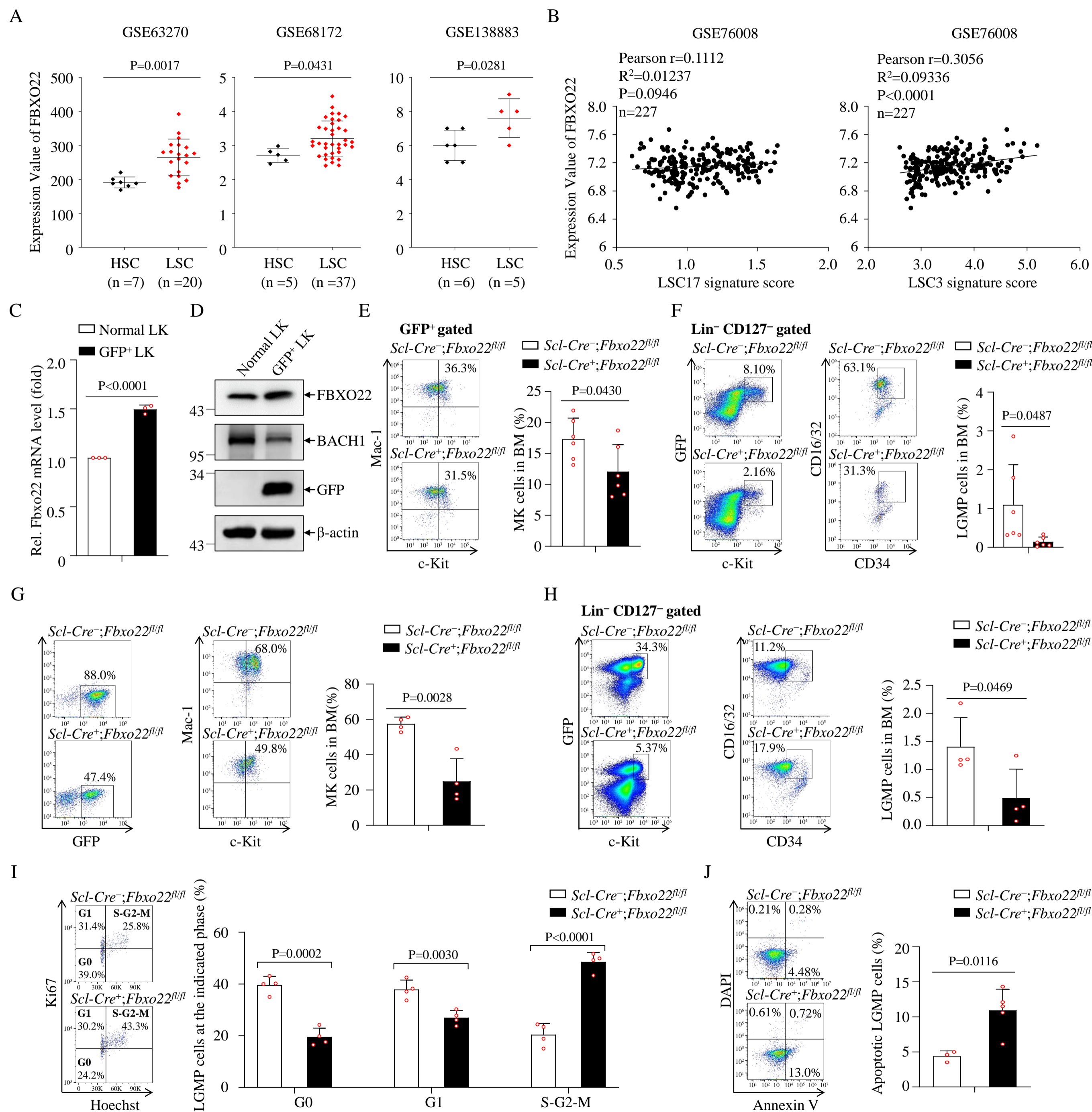

Supplement: Supplementary file 6 — Additional file 6. Figure S5. Loss of Fbxo22 impairs the function of LSCs. A FBXO22 mRNA expression level in HSC or LSC was analyzed in databases as indicated. In GSE63270, GSE138883 and GSE68172 datasets, HSCs were characterized, respectively, as Lin–CD34+CD38−CD90+CD45RA+, CD34+ and CD34+CD38− cells from bone marrow of healthy donors. LSCs were defined as CD34+CD38− in GSE63270 and CD34+ cells purified from bone marrow of AML patients in GSE138883 and GSE68172 datasets. B Pearson’s correlation between LSC17 (left) or LSC3 (right) signature score and FBXO22 mRNA expression level of AML patients from GSE76008 database. C, D FBXO22 expression level in Normal LKs or GFP+LKs was tested by qRT-PCR (C) or Western blot (D). E, F Flow cytometry plots (left) and the percentages of MKs (right, E) and LGMP (right, F) in BM from the primary recipients transplanted with Scl-Cre+;Fbxo22fl/fl and Scl-Cre−;Fbxo22fl/fl AML cells (n=6). G, H Flow cytometry plots (left) and the percentages of MKs (right, G) and LGMP (right, H) in BM from the secondary recipients transplanted with Scl-Cre+;Fbxo22fl/fl and Scl-Cre−;Fbxo22fl/fl AML cells (n=4). I, J Cell-cycle (I) or apoptosis (J) analysis of LGMP cells in BM from the secondary recipients transplanted with Scl-Cre+;Fbxo22fl/fl and Scl-Cre−;Fbxo22fl/fl AML cells (n=3-5). Error bars denote mean ± SD. Statistical significance was determined by two-tailed unpaired t test (A, C and E–J), and the P values were shown. All animal experiments were repeated at least twice with similar results [file 13045_2023_1400_MOESM6_ESM.pdf]

## Supplementary Figure S6

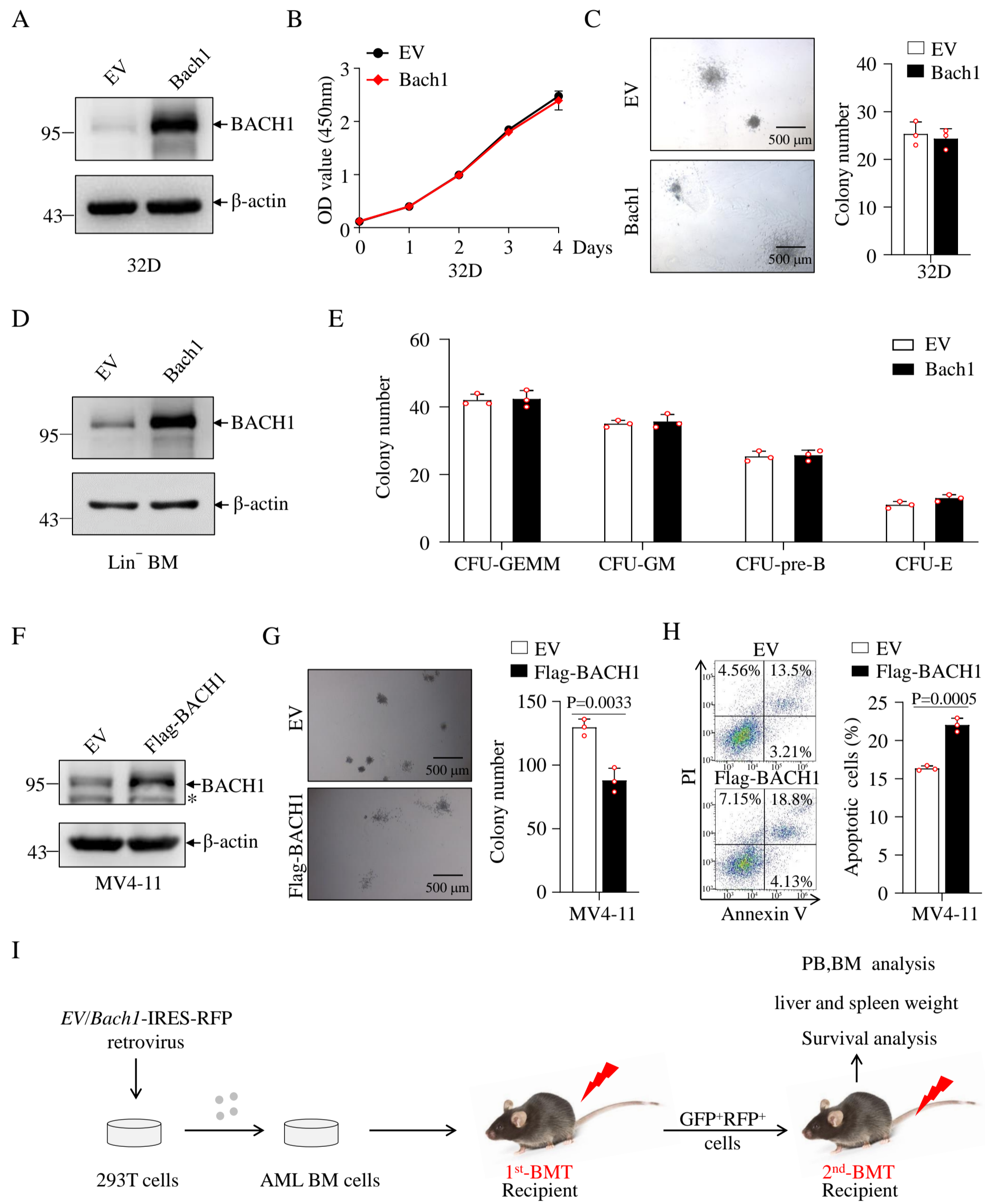

Supplement: Supplementary file 8 — Additional file 8. Figure S6. BACH1 suppresses MLLr AML progression. A, B 32D cells infected with EV or Bach1 and indicated proteins were immunoblotted with β-actin as loading control (A), cell proliferation was examined by CCK-8 assay at the indicated days (B). C Colony-forming assay (left) for the indicated 32D cells, and colony numbers at day 7 after plating were counted (right, n=3). D, E Lin− BM cells from wild-type mice infected with EV or Bach1 and indicated proteins were immunoblotted with β-actin as loading control (D), cells were seeded in indicated methylcellulose medium for the quantification of CFU-GEMM, CFU-GM, CFU-pre-B and CFU-E colonies (E, n=3). F MV4-11 cells were infected with Flag-tagged BACH1 or EV, indicated proteins were immunoblotted with β-actin as loading control. The asterisk represents a nonspecific band. G Representative images (left) of colonies and colony numbers (right, n=3) formed by MV4-11 cells infected with Flag-tagged BACH1 or EV. H Apoptosis analysis of MV4-11 cells infected with Flag-tagged BACH1 or EV. I Schematic strategy of evaluation of the in vivo effect of Bach1 overexpression in mouse AML cells. Error bars denote mean ± SD. Statistical significance was determined by two-tailed unpaired t test (C, E and G, H), two-way ANOVA (B) and the P values were shown. All experiments were repeated at least three times with similar results [file 13045_2023_1400_MOESM8_ESM.pdf]
