# Supplementary material for: FBXO22 promotes leukemogenesis by targeting BACH1 in MLL-rearranged acute myeloid leukemia
Source: J Hematol Oncol. 2023 Feb 11;16:9. doi: 10.1186/s13045-023-01400-0 (PMC9922468; doi:10.1186/s13045-023-01400-0)
Supplement: Supplementary file 9 — Additional file 9. Uncropped Western blots [file 13045_2023_1400_MOESM9_ESM.pdf]

Uncropped Western blots

Fig. 1B

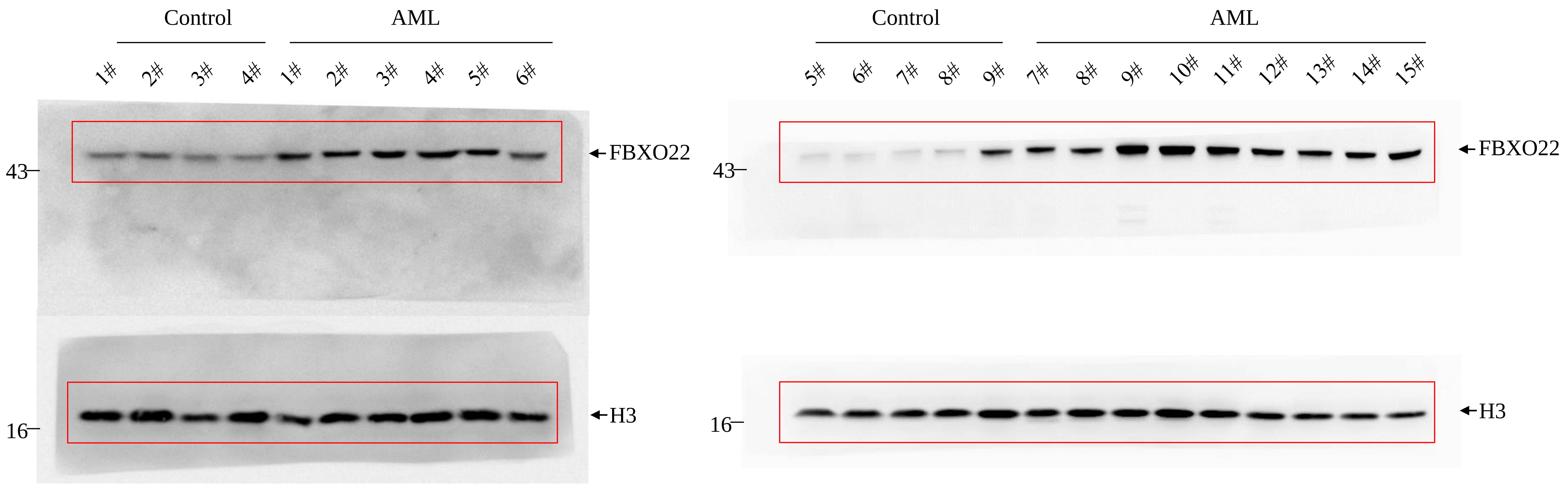

Fig. 1C

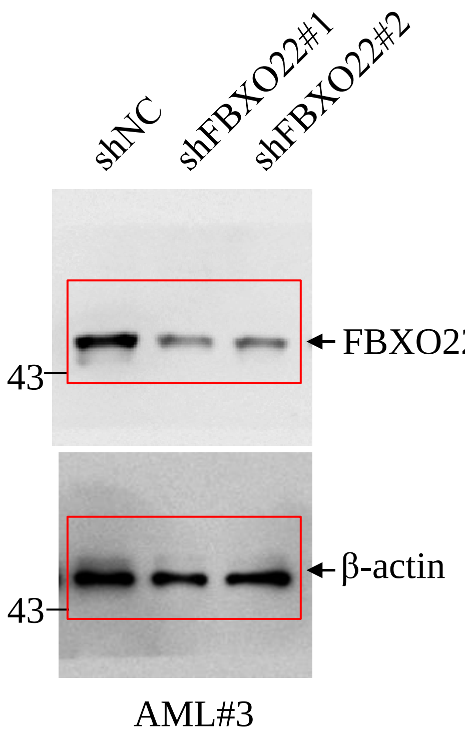

Fig. 1K

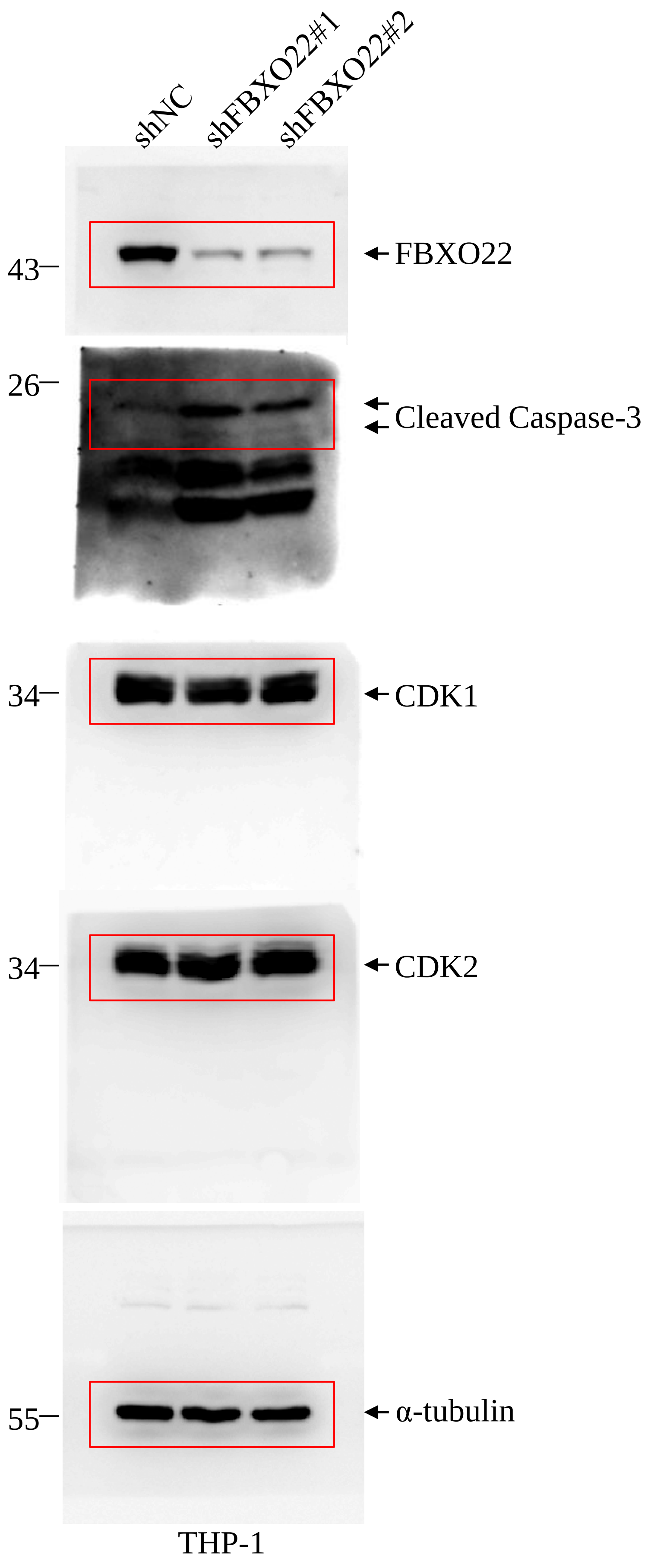

Fig. 1L

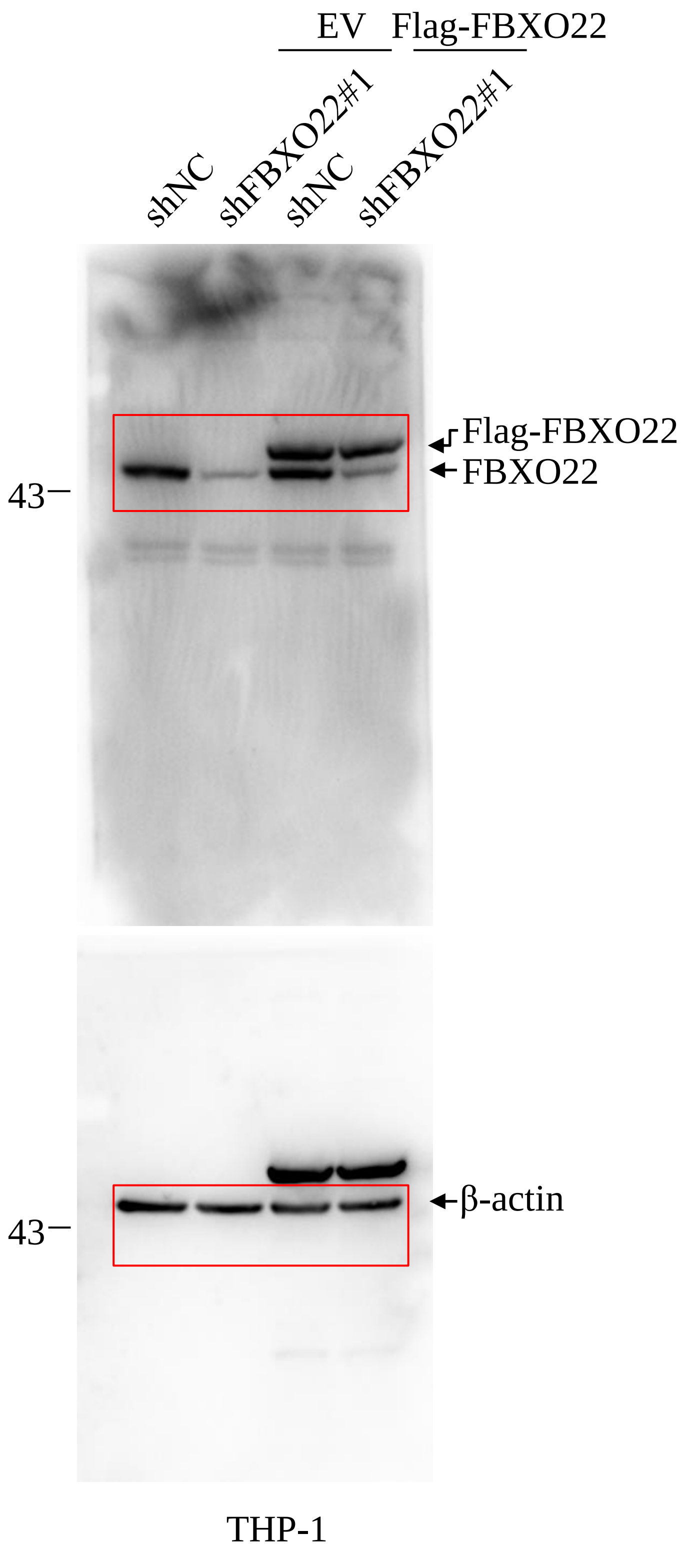

Fig. F

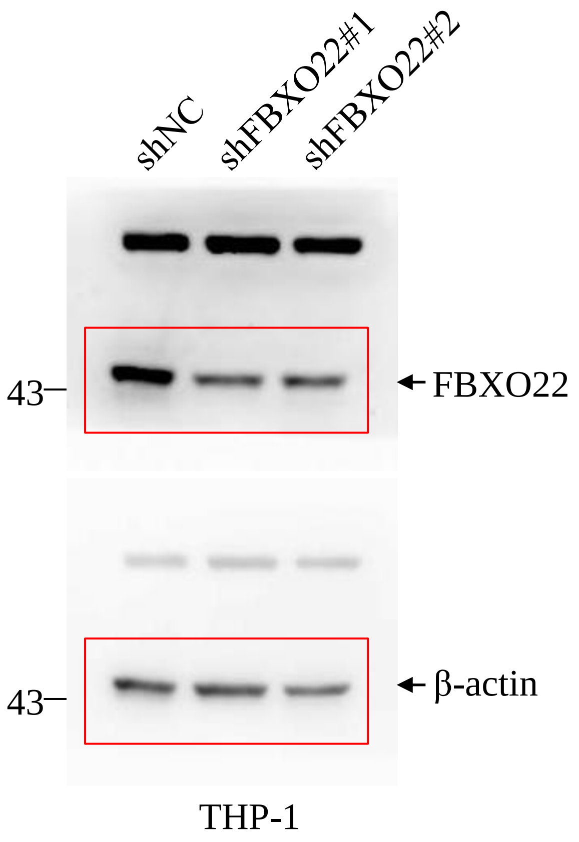

Uncropped Western blots

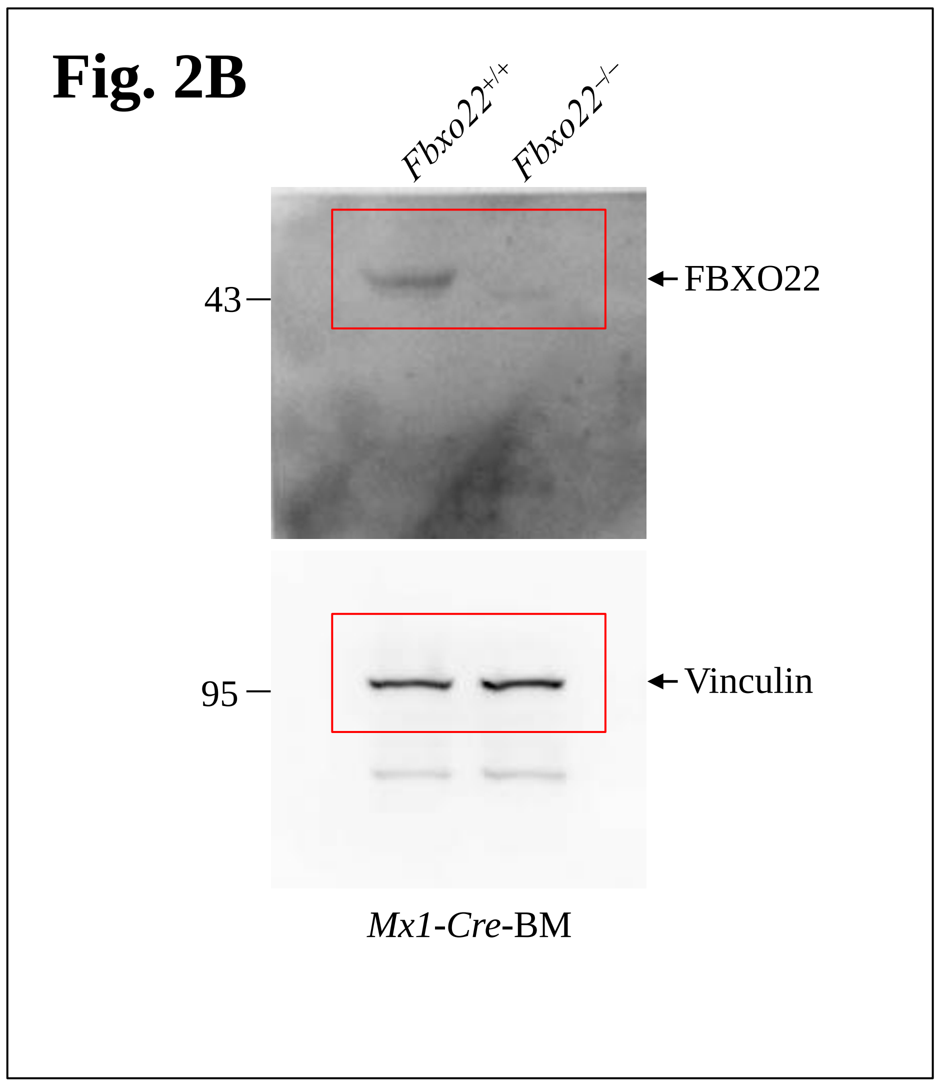

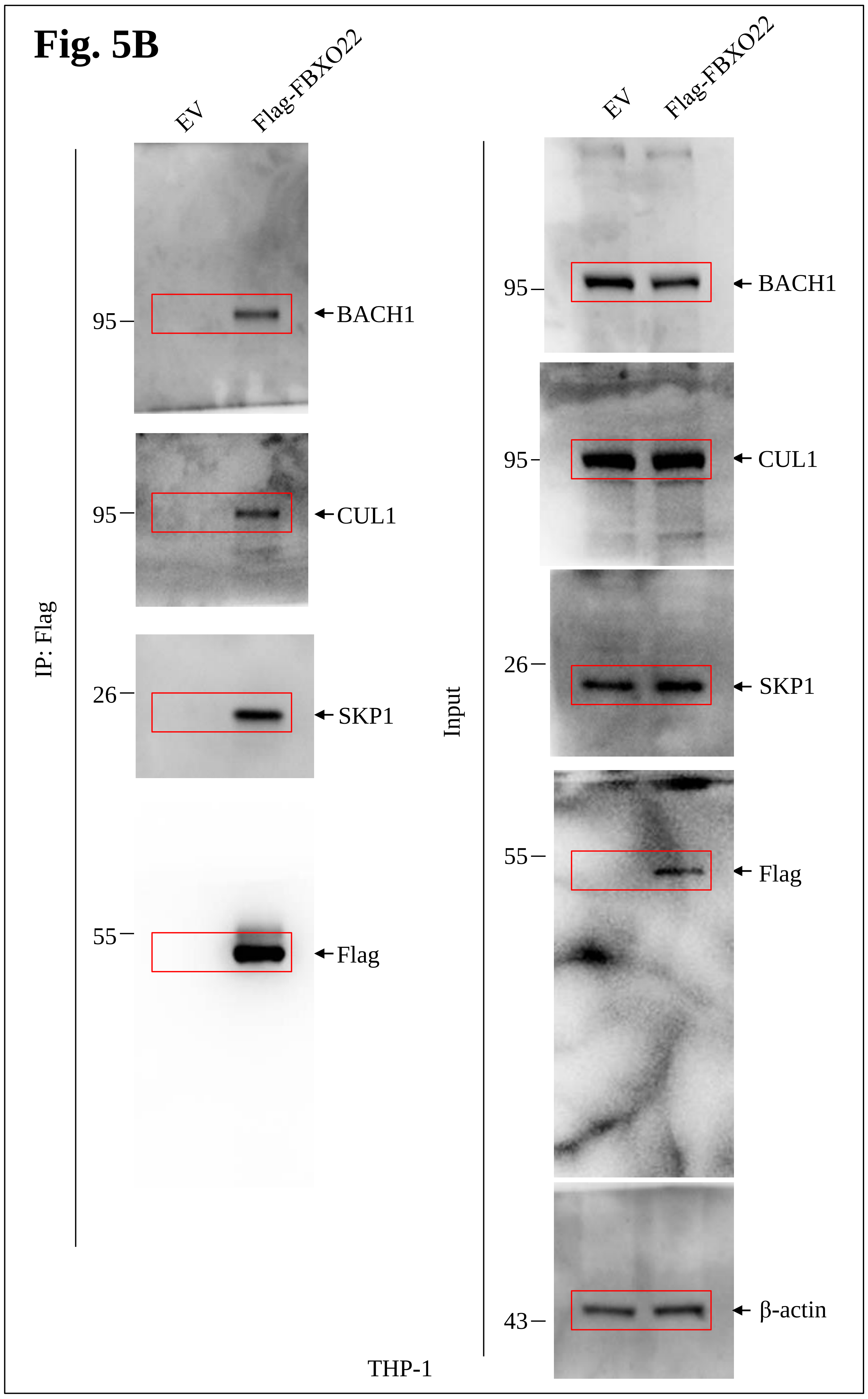

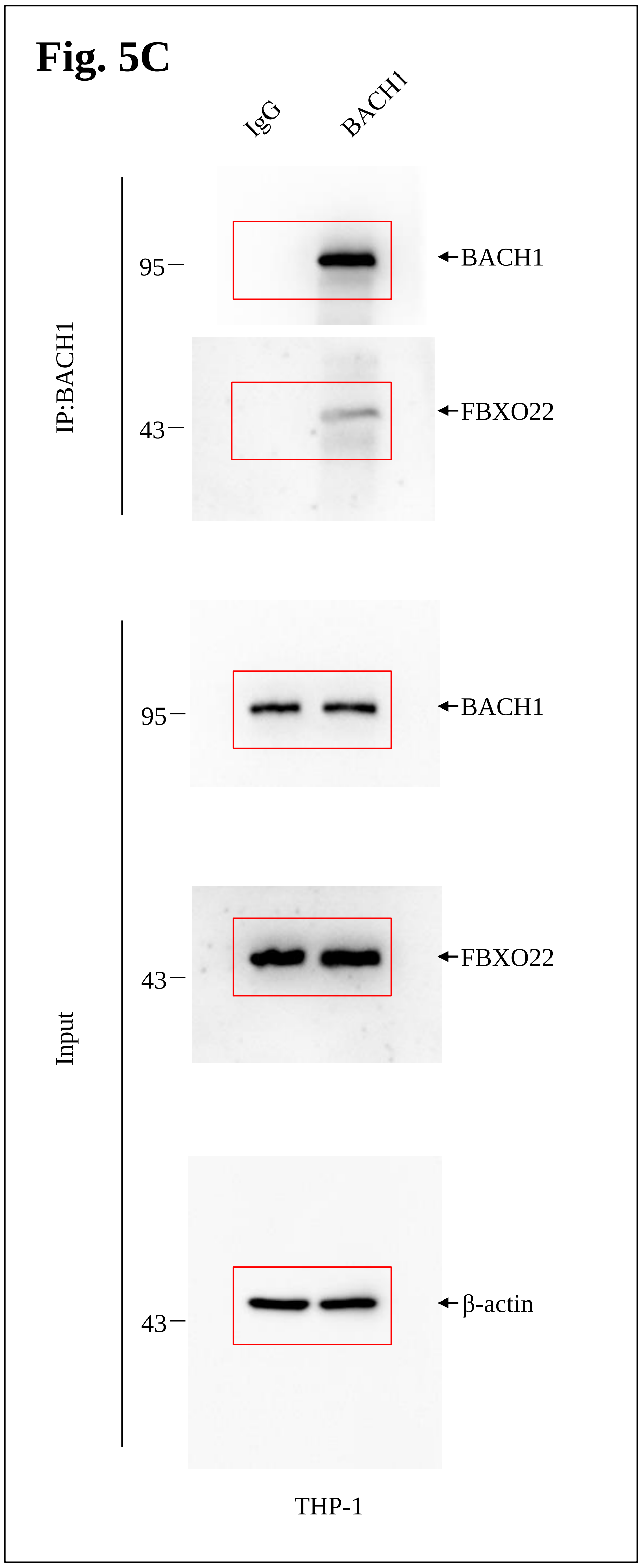

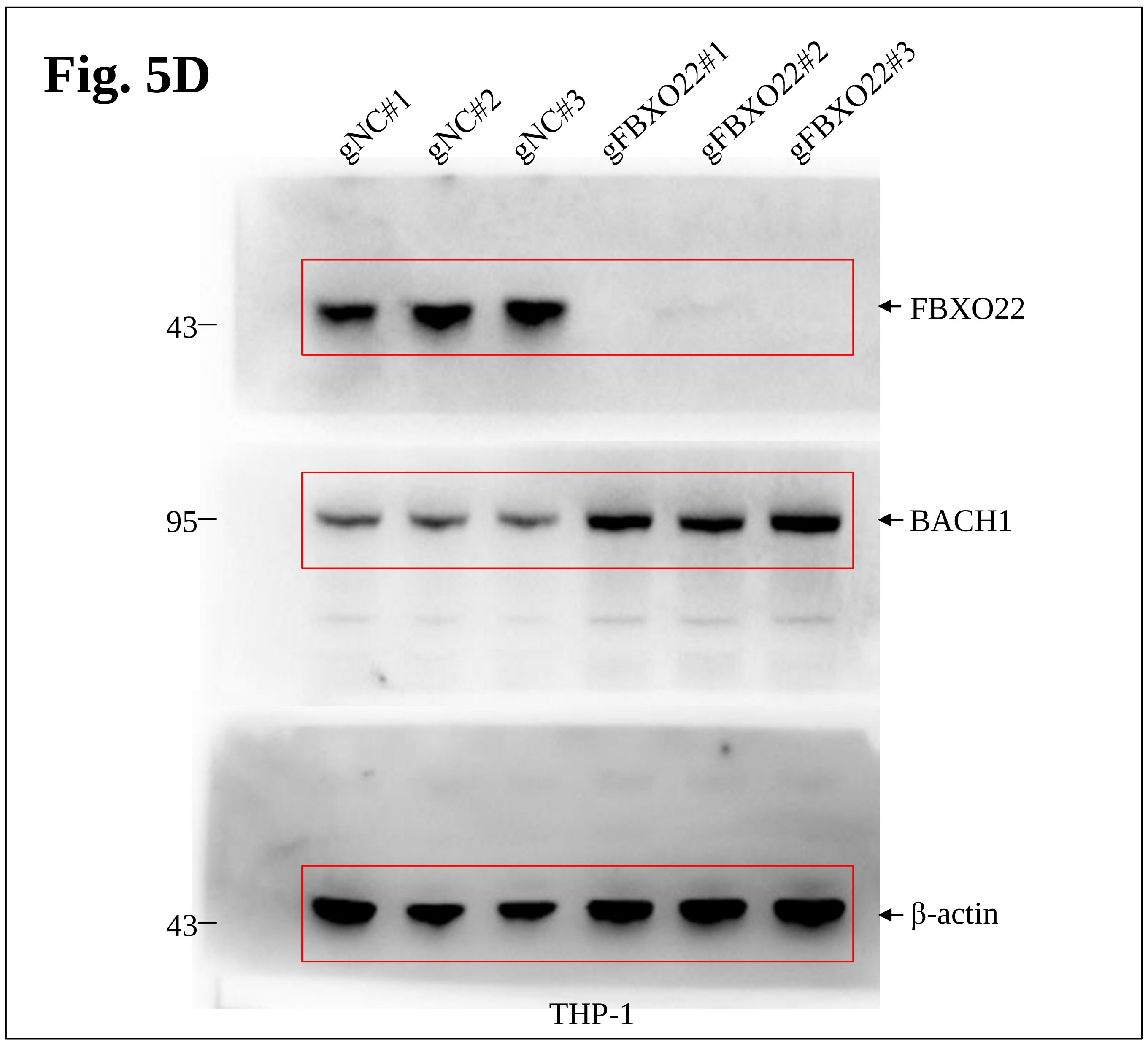

Uncropped Western blots

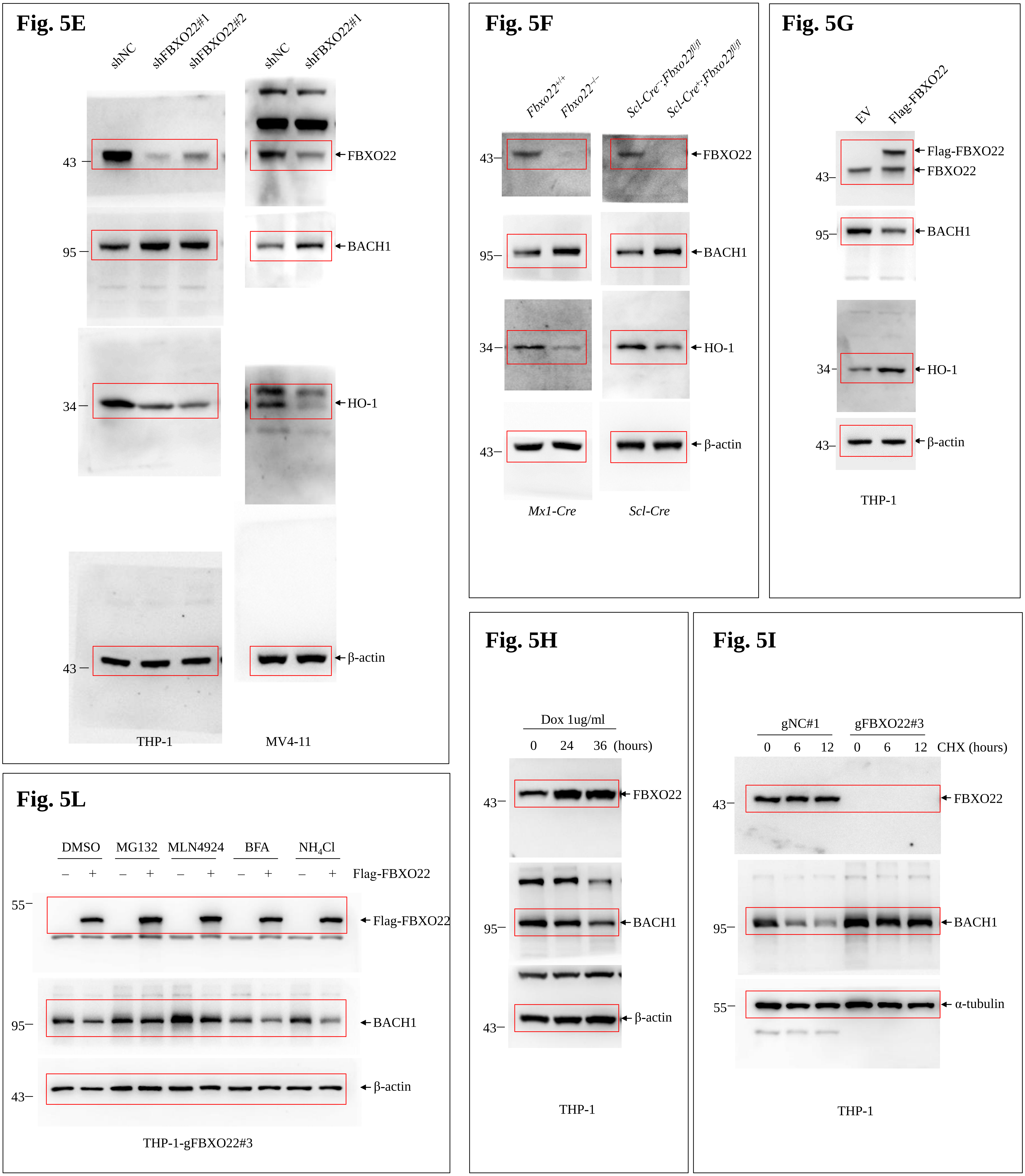

Uncropped Western blots

Fig. 5M

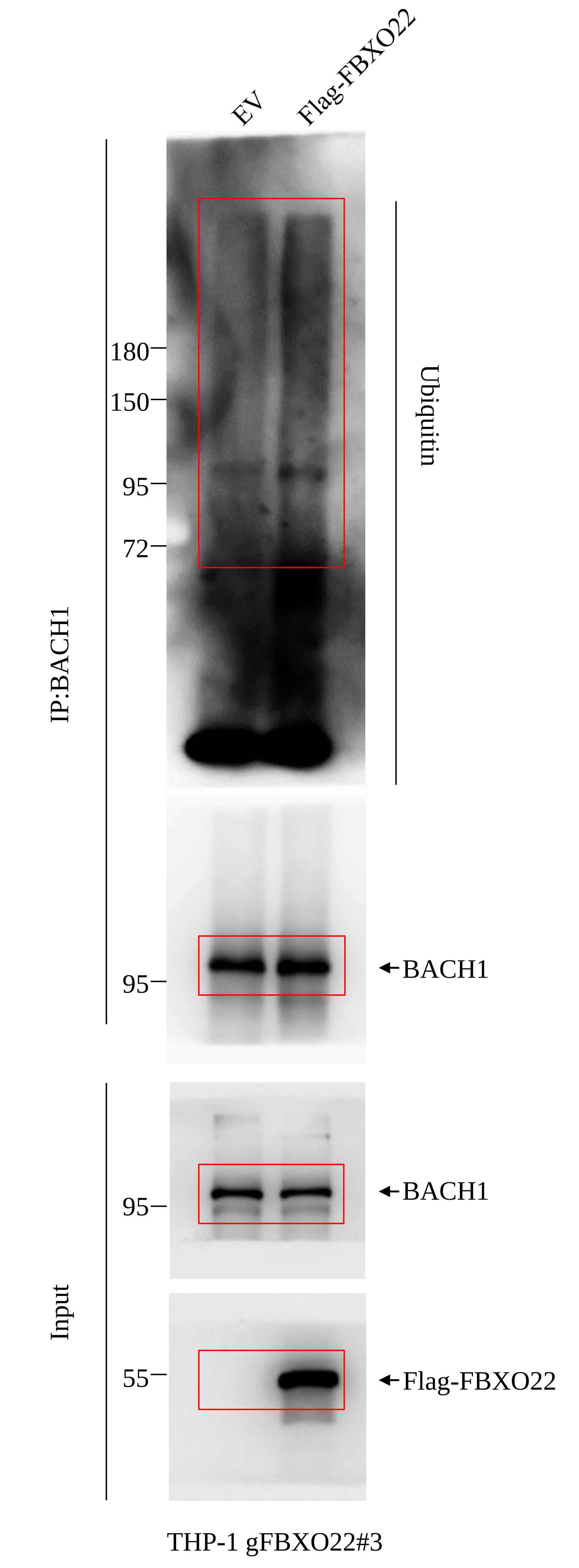

Fig. 5N

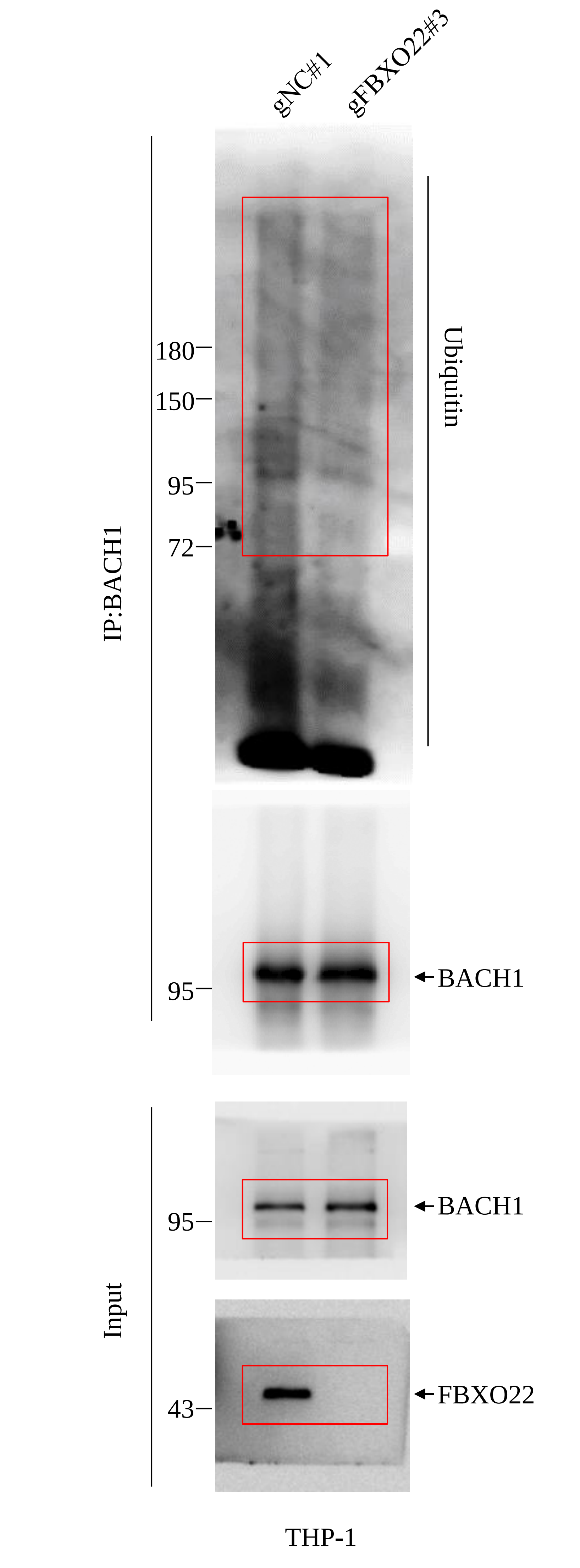

Fig. 6A

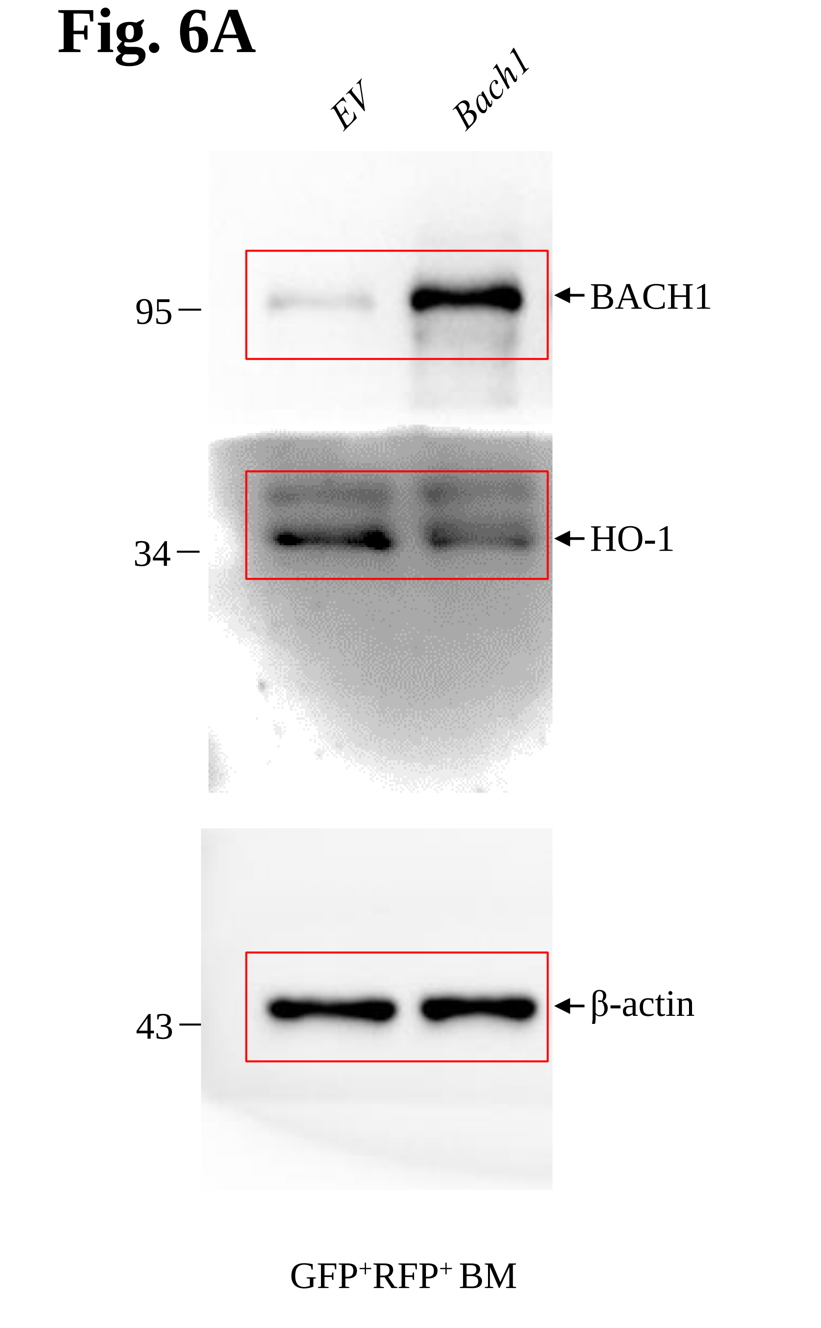

Fig. 7A

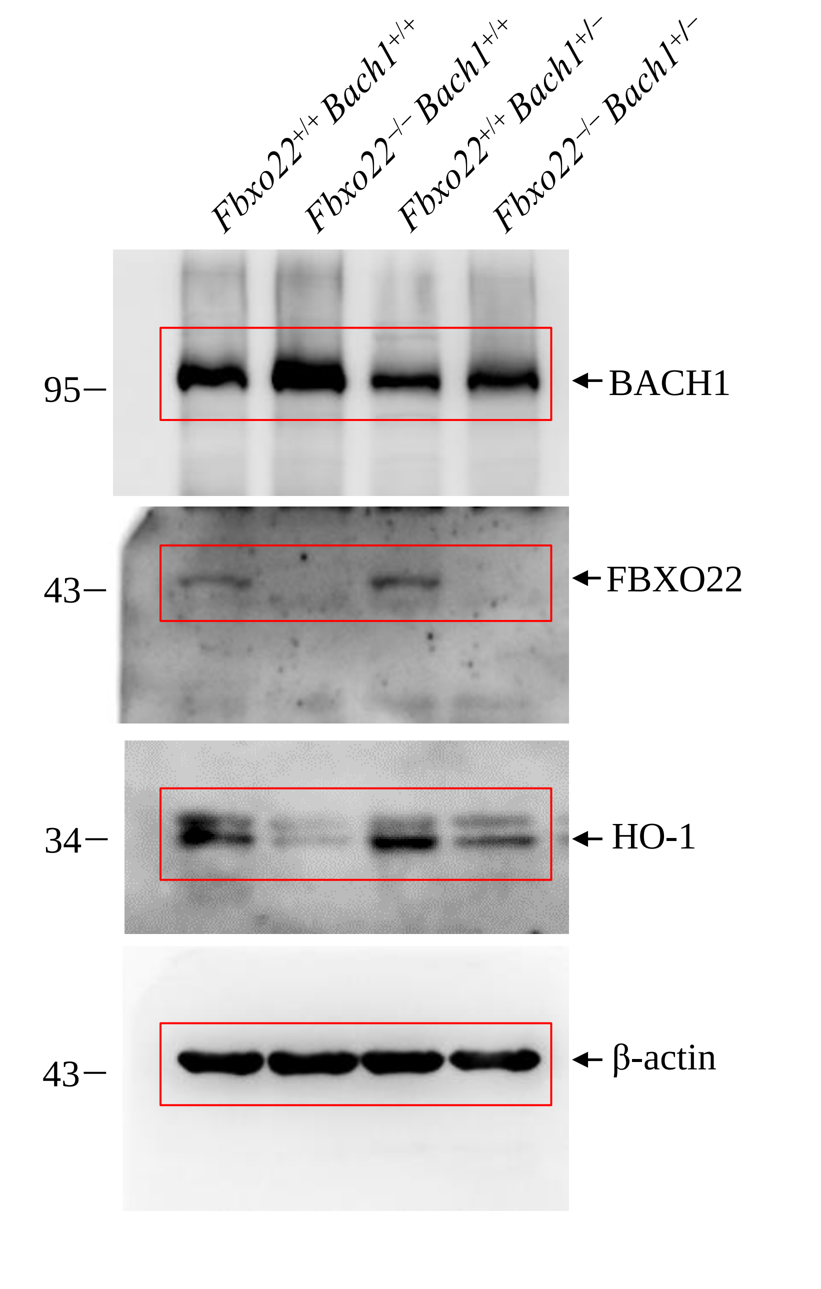

Uncropped Western blots

Fig. S1C

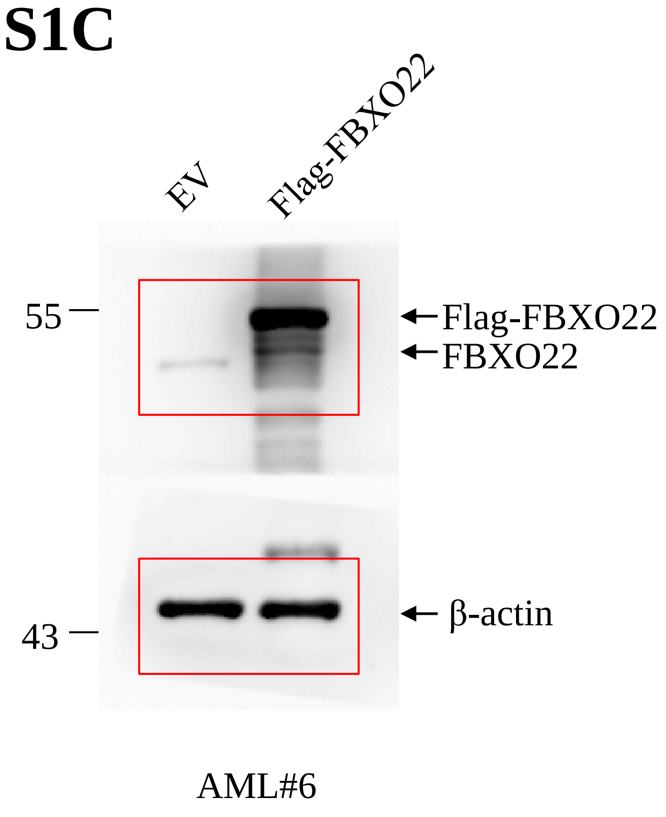

Fig. S1I

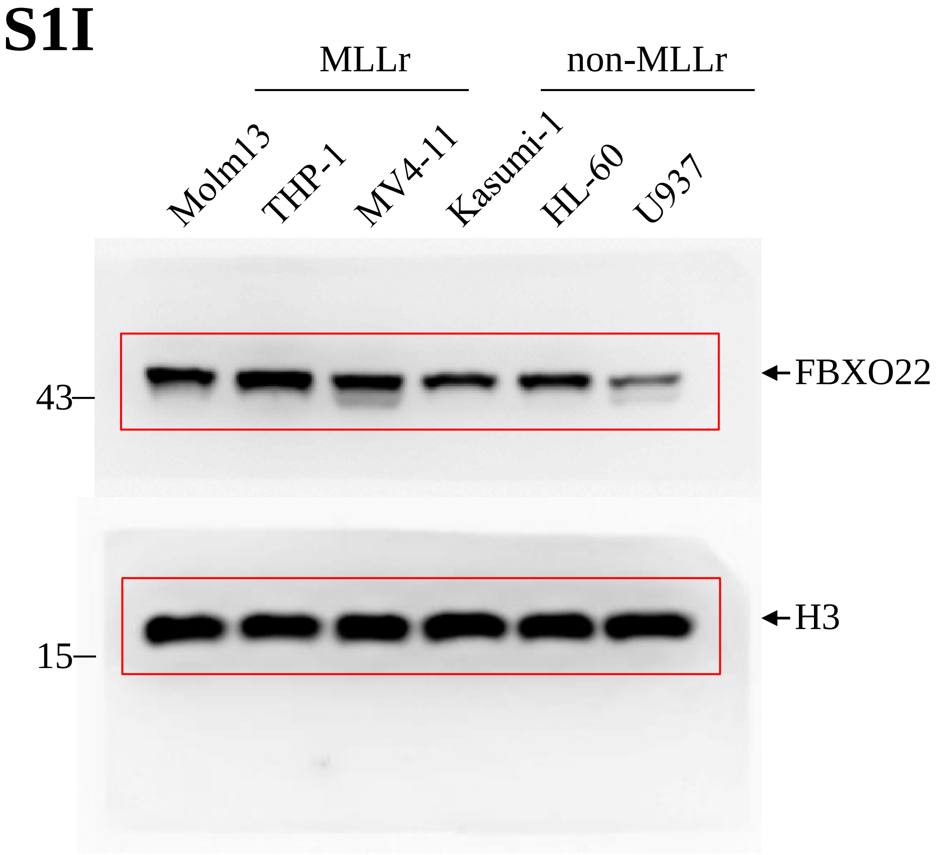

Fig. S2A

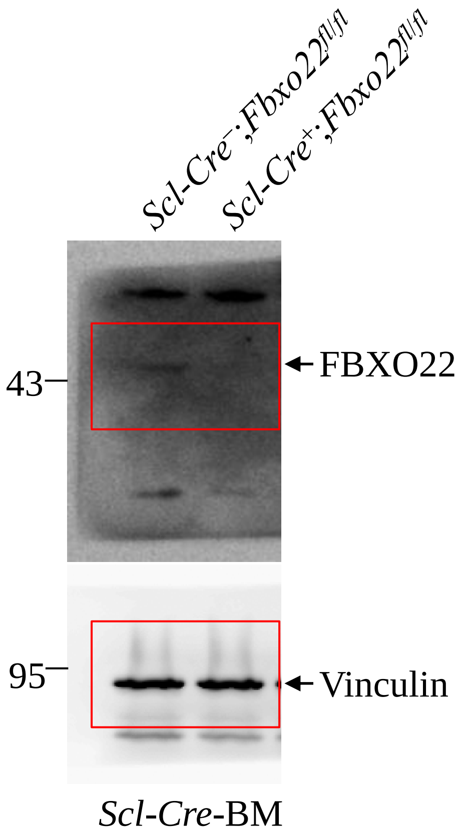

Fig. S3A

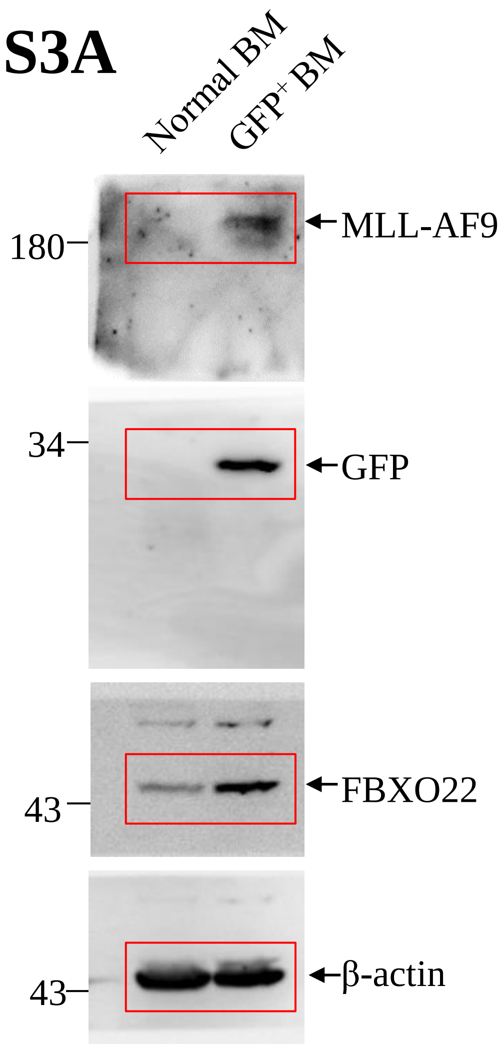

Fig. S5D

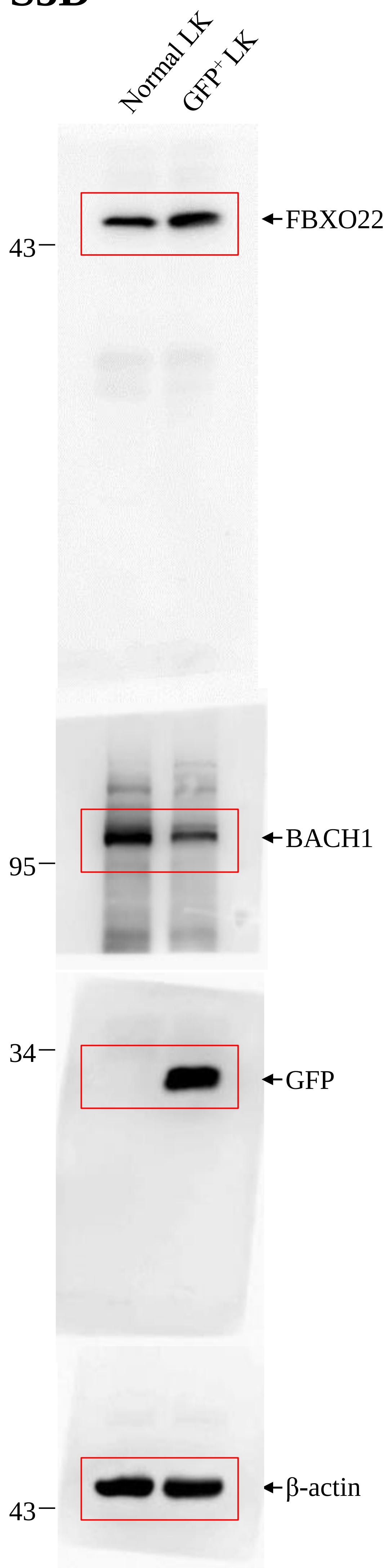

Fig. S2B

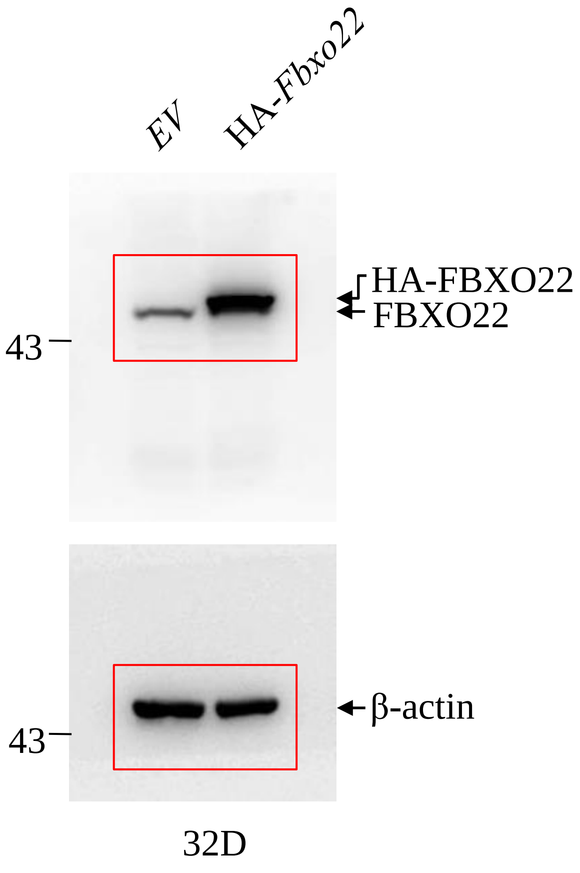

Fig. S4H

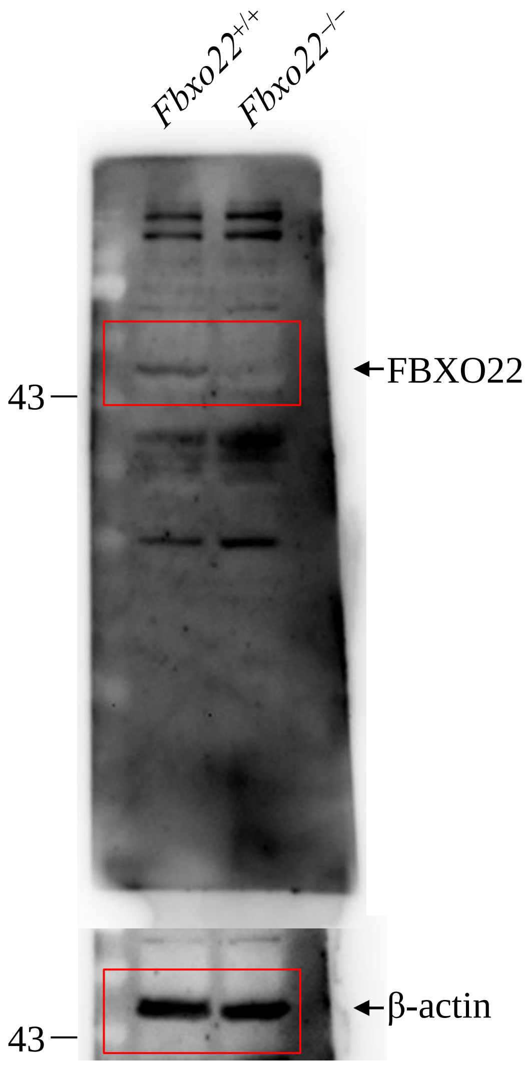

Uncropped Western blots

Fig. S6A

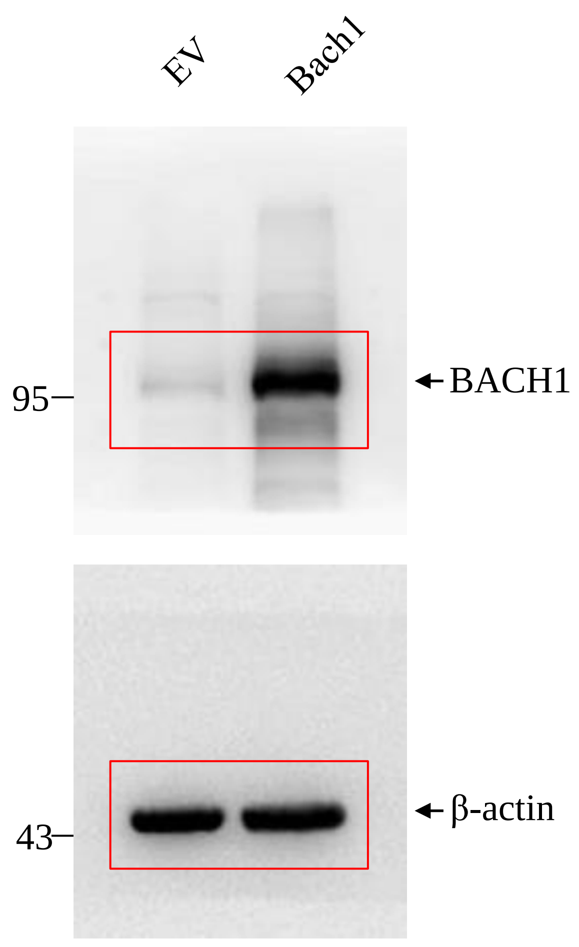

32D

Fig. S6D

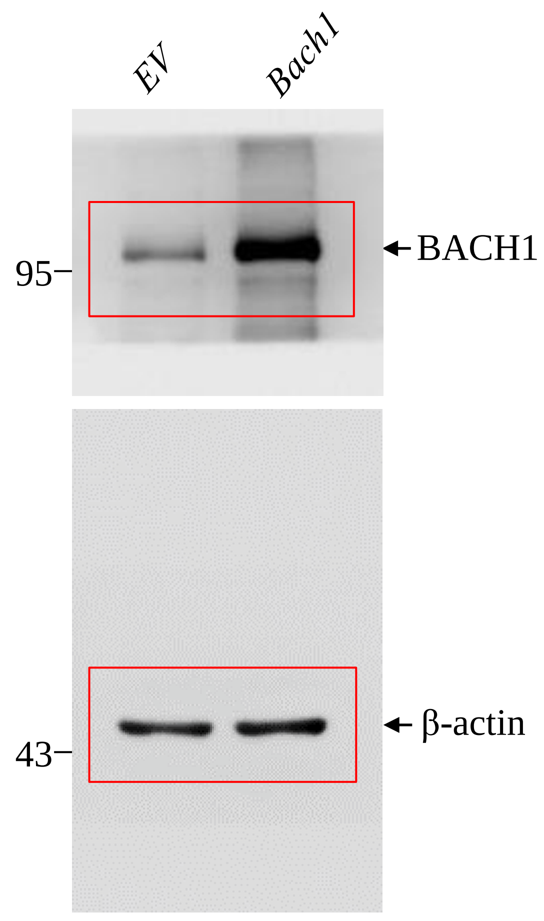

Lin$^-$ BM

Fig. S6F

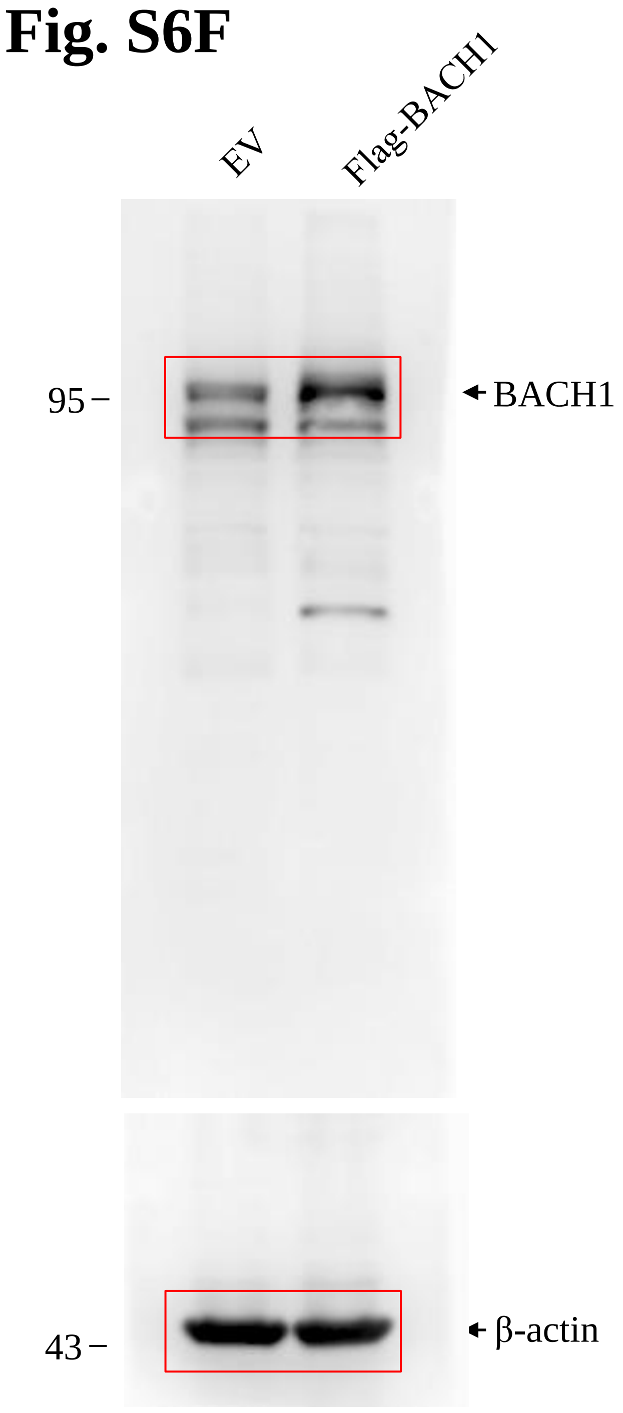

MV4-11
